# Supplementary material for: Food safety knowledge, attitudes, and practices among Jordanian women handling food at home during COVID-19 pandemic
Source: PLoS One. 2023 Jul 7;18(7):e0288323. doi: 10.1371/journal.pone.0288323 (PMC10328301; doi:10.1371/journal.pone.0288323)
Supplement: Attachment — Submitted filename: PONE-D-22-35672 revised.pdf [file pone.0288323.s001.pdf]

PLOS ONE

Food safety literacy among women food handlers at homes in Jordan during the COVID-19 pandemic

--Manuscript Draft--

**Manuscript Number:**	PONE-D-22-35672
**Article Type:**	Research Article
**Full Title:**	Food safety literacy among women food handlers at homes in Jordan during the COVID-19 pandemic
**Short Title:**	Food safety literacy among women food handlers at homes in Jordan
**Corresponding Author:**	Tareq Osaili<br>Jordan University of Science and Technology Faculty of Agriculture<br>Irbid, JORDAN
**Keywords:**	COVID-19, Personal Hygiene, Food Handling, Cross-Contamination, Foodborne Diseases, Households
**Abstract:**	Concerns over food safety during a coronavirus pandemic (COVID-19) have sparked worldwide interest. Food handlers at homes are the final line of defense in the food safety chain in reducing foodborne diseases. The present study sought to investigate the knowledge, attitudes, and practices (KAP) of women food handlers in Jordan. Furthermore, in order to investigate the effect of the COVID-19 epidemic on food handlers in terms of food safety KAP. The study was a cross-sectional survey. One thousand one hundred twenty-six respondents completed a food safety questionnaire during the COVID-19 epidemic. With a mean score of 22.1 out of 42 points, the results showed that the subjects had insufficient knowledge, negative attitudes, and incorrect practices with regard to food safety. The respondents demonstrated a high level of knowledge, attitudes, and practices in the "personal hygiene" and "cleaning and sanitation" areas (≥ 60.0%). The subjects' knowledge, attitudes, and practices in "contamination prevention," "health issues that would affect food safety," "symptoms of foodborne illnesses," "safe storage, thawing, cooking, keeping, and reheating of foods," and "COVID-19" areas were all low (< 60.0%). The correlations between participants' total food safety KAP scores and education, age, experience, region, and the COVID-19 pandemic's effects on food safety were statistically significant ($P \leq 0.05$). According to our information, this is the first study to investigate food safety knowledge, attitudes, and practices among Jordanian women food handlers during the COVID-19 epidemic. Food safety training courses for female food handlers at home are required.
**Order of Authors:**	Tasneem M. Al-Jaberi
	Anas A. Al-Nabulsi
	Tareq Osaili
	Amin N. Olaimat
	Sawsan Mutlaq
**Additional Information:**	
**Question**	**Response**
**Financial Disclosure**<br><br>Enter a financial disclosure statement that describes the sources of funding for the work included in this submission. Review the submission guidelines for detailed requirements. View published research articles from *PLOS ONE* for specific examples.	The author(s) received no specific funding for this work.

Powered by Editorial Manager® and ProduXion Manager® from Aries Systems Corporation

This statement is required for submission and **will appear in the published article** if the submission is accepted. Please make sure it is accurate.<br><br>**Unfunded studies**<br>Enter: *The author(s) received no specific funding for this work.*<br><br>**Funded studies**<br>Enter a statement with the following details:<br>• Initials of the authors who received each award<br>• Grant numbers awarded to each author<br>• The full name of each funder<br>• URL of each funder website<br>• Did the sponsors or funders play any role in the study design, data collection and analysis, decision to publish, or preparation of the manuscript?<br>• **NO** - Include this sentence at the end of your statement: *The funders had no role in study design, data collection and analysis, decision to publish, or preparation of the manuscript.*<br>• **YES** - Specify the role(s) played.<br><br>* typeset	
**Competing Interests**<br><br>Use the instructions below to enter a competing interest statement for this submission. On behalf of all authors, disclose any competing interests that could be perceived to bias this work—acknowledging all financial support and any other relevant financial or non-financial competing interests.<br><br>This statement is required for submission and **will appear in the published article** if the submission is accepted. Please make sure it is accurate and that any funding sources listed in your Funding Information later in the submission form are also declared in your Financial Disclosure statement.<br><br>View published research articles from	The authors have declared that no competing interests exist.

Powered by Editorial Manager® and ProduXion Manager® from Aries Systems Corporation

PLOS ONE for specific examples.<br><br>**NO authors have competing interests**<br><br>Enter: *The authors have declared that no competing interests exist.*<br><br>**Authors with competing interests**<br><br>Enter competing interest details beginning with this statement:<br><br>*I have read the journal's policy and the authors of this manuscript have the following competing interests: [insert competing interests here]*<br><br>* typeset	
**Ethics Statement**<br><br>Enter an ethics statement for this submission. This statement is required if the study involved:<br><br>• Human participants<br>• Human specimens or tissue<br>• Vertebrate animals or cephalopods<br>• Vertebrate embryos or tissues<br>• Field research<br><br>Write "N/A" if the submission does not require an ethics statement.<br><br>General guidance is provided below. Consult the submission guidelines for detailed instructions. **Make sure that all information entered here is included in the Methods section of the manuscript.**	The study and the protocol were approved by the Institutional Review Board (IRB) at Jordan University of Science and Technology in session number 29/146/2021.

**Format for specific study types**<br><br>**Human Subject Research (involving human participants and/or tissue)**<br>• Give the name of the institutional review board or ethics committee that approved the study<br>• Include the approval number and/or a statement indicating approval of this research<br>• Indicate the form of consent obtained (written/oral) or the reason that consent was not obtained (e.g. the data were analyzed anonymously)<br><br>**Animal Research (involving vertebrate animals, embryos or tissues)**<br>• Provide the name of the Institutional Animal Care and Use Committee (IACUC) or other relevant ethics board that reviewed the study protocol, and indicate whether they approved this research or granted a formal waiver of ethical approval<br>• Include an approval number if one was obtained<br>• If the study involved *non-human primates*, add *additional details* about animal welfare and steps taken to ameliorate suffering<br>• If anesthesia, euthanasia, or any kind of animal sacrifice is part of the study, include briefly which substances and/or methods were applied<br><br>**Field Research**<br><br>Include the following details if this study involves the collection of plant, animal, or other materials from a natural setting:<br>• Field permit number<br>• Name of the institution or relevant body that granted permission	
**Data Availability**<br><br>Authors are required to make all data underlying the findings described fully available, without restriction, and from the time of publication. PLOS allows rare exceptions to address legal and ethical concerns. See the PLOS Data Policy and FAQ for detailed information.	Yes - all data are fully available without restriction

A Data Availability Statement describing where the data can be found is required at submission. Your answers to this question constitute the Data Availability Statement and **will be published in the article**, if accepted.<br><br>**Important:** Stating 'data available on request from the author' is not sufficient. If your data are only available upon request, select 'No' for the first question and explain your exceptional situation in the text box.<br><br>Do the authors confirm that all data underlying the findings described in their manuscript are fully available without restriction?	
**Describe where the data may be found in full sentences. If you are copying our sample text, replace any instances of XXX with the appropriate details.**<br><br>• If the data are **held or will be held in a public repository**, include URLs, accession numbers or DOIs. If this information will only be available after acceptance, indicate this by ticking the box below. For example: *All XXX files are available from the XXX database (accession number(s) XXX, XXX.).*<br>• If the data are all contained **within the manuscript and/or Supporting Information files**, enter the following: *All relevant data are within the manuscript and its Supporting Information files.*<br>• If neither of these applies but you are able to provide **details of access elsewhere**, with or without limitations, please do so. For example:<br><br>*Data cannot be shared publicly because of [XXX]. Data are available from the XXX Institutional Data Access / Ethics Committee (contact via XXX) for researchers who meet the criteria for access to confidential data.*<br><br>*The data underlying the results presented in the study are available from (include the name of the third party*	All relevant data are within the manuscript and its Supporting Information files.

Powered by Editorial Manager® and ProduXion Manager® from Aries Systems Corporation

and contact information or URL).<br>• This text is appropriate if the data are owned by a third party and authors do not have permission to share the data.<br><br>* typeset	
Additional data availability information:	Tick here if your circumstances are not covered by the questions above and you need the journal’s help to make your data available.

Powered by Editorial Manager® and ProduXion Manager® from Aries Systems Corporation

Manuscript

Click here to access/download;Manuscript;Food safety KAP among households 13.1.2023.docx

Food safety literacy among women food handlers at homes in Jordan during the COVID-19 pandemic

Tasneem M. Al-Jaberi[1], Anas A. Al-Nabulsi[1], Tareq M. Osaili[1,2 *], Amin N. Olaimat[3], Sawsan Mutlaq[1]

[1] Department of Nutrition and Food Technology, Faculty of Agriculture, Jordan University of Science and Technology, P.O. Box 3030, Irbid 22110, Jordan. tmaljaberi19@agr.just.edu.jo, anas_nabulsi@just.edu.jo , tosaili@just.edu.jo , shmutlaq17@agr.just.edu.jo

[2] Department of Clinical Nutrition and Dietetics, College of Health Sciences, University of Sharjah, P. O. Box 27272, Sharjah, United Arab Emirates.

[3] Department of Clinical Nutrition and Dietetics, Faculty of Applied Medical Sciences, The Hashemite University, Zarqa 13133, Jordan. aminolaimat@hu.edu.jo

*** Corresponding author:**

Prof. Tareq M. Osaili

E-mail address : tosaili@just.edu.jo

Abstract

Concerns over food safety issues during a coronavirus pandemic (COVID-19) have sparked worldwide interest. Food handlers, being part of a farm to fork of the food safety chain, at homes, consist the final line of defensein in reducing foodborne diseases. The present study sought to investigate the knowledge, attitudes, and practices (KAP) of women food handlers in Jordan. Furthermore, in order to investigate the effect of the COVID-19 epidemic on food handlers in terms of food safety KAP. The study was a cross-sectional survey. One thousand one hundred twenty-six respondents completed a food safety questionnaire during the COVID-19 epidemic. With a mean score of 22.1 out of 42 points, the results showed that the subjects had insufficient knowledge, negative attitudes, and incorrect practices with regard to food safety. The respondents demonstrated a high level of knowledge, attitudes, and practices in the "personal hygiene" and "cleaning and sanitation" areas (≥ 60.0%). The subjects' knowledge, attitudes, and practices in "contamination prevention," "health issues that would affect food safety," "symptoms of foodborne illnesses," "safe storage, thawing, cooking, keeping, and reheating of foods," and "COVID-19" areas were all low (< 60.0%). The correlations between participants' total food safety KAP scores and education, age, experience, region, and the COVID-19 pandemic's effects on food safety were statistically significant (P ≤ 0.05). According to our information, this is the first study to investigate food safety knowledge, attitudes, and practices among Jordanian women food handlers during the COVID-19 epidemic. Food safety training courses for female food handlers at home are required.

Keywords:

COVID-19, Personal Hygiene, Food Handling, Cross-Contamination, Foodborne Diseases, Households

1. Introduction

Food safety is a dilemma for consumers, food providers, and governmental agencies worldwide. Foodborne pathogen-contaminated food has always been seen as a concern for global public health owing to the diseases and disorders that may ensue from its ingestion. The World Health Organization (WHO) predicts that contaminated food causes almost 600 million disease cases and 420,000 deaths globally each year due to bacteria, viruses, parasites, poisons, and chemicals [1]. In addition, the Eastern Mediterranean has the third-highest rate of foodborne diseases [1]. The incidence of foodborne infections in Jordan is unclear, although research has evaluated the burden of selected foodborne pathogens for one year. Jordan has recorded at least 6612 Salmonella, 16,260 Shigella, and 6912 Brucella outbreaks every year [2]. Meanwhile, in Jordan, a food poisoning outbreak has claimed the life of a toddler and affected over 800 individuals. They all had low-cost shawarma from a restaurant outside Amman [3].

According to the WHO, foodborne illnesses are caused by various factors, including cross-contamination, poor storage conditions, insecure food sources, food mishandling, poor personal hygiene, and improper cooking [4]. Foodborne outbreaks can occur in any place, including food handling, storage, and preparation [5]. Previous studies have found that food consumed at home is the leading source of foodborne illness outbreaks among consumers but that many instances are often misdiagnosed or underreported [6]. Furthermore, according to the European Union's overview study, 40.5% of foodborne outbreaks happen at home [7]. To prevent foodborne illness at home, food handlers, especially women, need to have adequate knowledge and good practices in food preparation, handling, and storage to minimize cross-contamination with hazards [8]. Multiple survey studies have indicated that home food handlers lack enough knowledge about food safety [6,8,9,10,11].

There are few local studies examining food safety on a national scale in Jordan. Like other Middle Eastern neighbors, Jordan's home kitchen is a prominent place for food preparation [12]. The food safety knowledge of Jordanian people was assessed by Okour, Alzein, & Saadeh in 2020 [13]. They discovered that food consumers in Jordan lack knowledge.

Concerns about food safety during a coronavirus pandemic (COVID-19) have inspired a global effort to adopt hygienic measures. In the COVID-19 period, food safety was the most impacted part of the food system [14]. The coronavirus disease (COVID-19) was considered to be a pandemic on March 11, 2020, by WHO, which is a highly contagious disease among countries and from person to person through respiratory droplets, which is transmitted from infected people via sneezing, coughing, or simply by breathing and talking [15]. Most infected people with coronavirus suffer from flu-like symptoms such as fever and coughing five days after infection, but in some cases, they suffer from a sore throat, loss of taste or scent, and muscle aches. It can also lead to acute respiratory complications and death in the worst-case situation [16]. There is presently no indication that COVID-19 can be transmitted through food. Coronaviruses cannot proliferate on the surface of food packages because they require a living animal or human host to reproduce and thrive. Food packages do not need to be sanitized, but hands should be disinfected after touching them and before eating [17].

During the COVID-19 pandemic, food systems confront significant obstacles in ensuring safe food handling and consumption along the food chain from farm to fork under the jurisdiction of relevant government agencies [18]. Therefore, there is an urgent need to examine knowledge, attitudes, and practices (KAP) regarding food safety during the era of the COVID-19 pandemic, in which many survey studies were conducted to investigate KAP among different sectors

worldwide, with very few studies in Jordan. According to the findings, KAP has improved during this public health crisis [19, 20, 21, 22, 23].

Most countries resorted to applying home quarantine to stop the spread of COVID-19, which increased homemade food preparation [24]. According to the societal culture in Jordan, women are responsible for food preparation and handling at home. As a result, the purpose of this research was to evaluate the food safety KAP among women food handlers at home during the era of the COVID-19 pandemic in Jordan. Also, to investigate the impacts of the COVID-19 pandemic on food safety KAP.

2. Materials and methods:

2.1. Research strategy:

The current study is a cross-sectional survey conducted during the era of the COVID-19 pandemic in Jordan. It relied on an online self-administered questionnaire to evaluate food safety KAP among women food handlers at home. Jordan University of Science and Technology's Deanship of Graduate Studies and Institutional Review Board (IRB) authorized the study and procedure in session number 29/146/2021.

2.2. Participants:

The estimated number of households in Jordan at the end of 2020 was 2206129 based on the department of statistics [25]. Thus, a sample size of at least 1039 people would be necessary to have a confidence level of 99.99%, as calculated by the "Sample Size Calculator," as equations shown below. So, a total of 1126 women food handlers who were 18 years old or older participated in the study and had the primary responsibility of food preparation at home. The participants were provided the required information regarding the study and gave their agreement to participate.

$$CI = p \pm z \times \sqrt{\frac{p(1-p)}{n}}$$

Where CI is Confidence Interval, z is z score, p is the population proportion and n is the sample size

$$Unlimited\ population: n = \frac{z^2 \times p\ (1-p)}{\varepsilon^2}$$

$$Finite\ population: n' = \frac{n}{1 + \frac{z^2 \times p(p-1)}{\varepsilon^2 N}}$$

Where z is the z score, ε is the margin of error, N is the population size and p is the population proportion.

2.3. Questionnaire design:

The survey tools were split into three parts: the first part included queries on the socio-demographic data of women food handlers (age, marital status, educational level, and years of experience). The second and third parts included questions on KAP on food safety and COVID-19 aspects, which were adapted from previous papers with some improvements [18, 26, 27]. Personal hygiene, cleaning and sanitation, cross-contamination prevention, health issues that would affect food safety, safe storage, thawing, cooking, keeping, and reheating of foods, sign and symptoms of foodborne illnesses, and COVID-19 KAP were the topics covered in these two parts.

2.4. Pilot study:

First step: The survey tools were created in both Arabic and English and were individually reviewed by food safety experts to authenticate survey content based on subject complexity and accurately assess what was meant to be assessed. Second step: For face-to-face validation of the questionnaire, ten people were chosen at random. The subjects were told to keep note of the time

it took them to fill out the questionnaire, how understandable the content was, and the language and vocabulary used. Food safety experts and academics examined and debated their replies to the questionnaire's instructions and content. In response to their feedback, minor tweaks and fixes were made to the questionnaire. The inquiries were multiple-choice and Likert scale, and they took around 15-20 minutes to fill out.

Third step: To assess the questionnaire's reliability, a pilot study was carried out on 30 women, who were subsequently requested not to fill out the final version of the survey. The internal consistency reliability approach was used to determine the extent to which the food safety KAP test is reliable in assessing what it is supposed to evaluate. In terms of general internal reliability, the questionnaire was reliable (Cronbach's Alpha = 0.71).

2.5. Distribution of questionnaires and response bias:

The questionnaire was sent across several platforms on social media to guarantee that each person had an equal likelihood of receiving a survey invitation. Since Not all people have a social media account that they can use to respond to online surveys, the investigators handed out additional questionnaires after calling individuals who did not have a social media account. Researchers kept the survey anonymous and worked on creating a questionnaire with neutrally framed questions that did not offer leading response possibilities to eliminate bias.

2.6. Statistical analysis:

The data was analyzed using the IBM SPSS Statistical Package for Social Sciences. For each variable, measures of dispersion (means, standard errors, frequencies, and percentages) were computed. A multivariate general linear model (GLM) was used to determine the association between subjects' general characteristics and the overall food safety KAP score. In addition, to figure out the correlation between the effect of the COVID-19 epidemic and subjects' KAP scores

for food safety, the nonparametric Kruskal-Wallis test was done. Also, to determine the connection between major food safety aspect ratings and improvements in participants' food safety measures during COVID-19.

Moreover, a multivariable binary logistic regression analysis was performed using the entry approach to discover variables that were independently related to a high level of food safety KAP. The characteristics of respondents were factored into the regression models to assess their relationship with the overall KAP level for food safety. Results with a P-value of < 0.05 were deemed statistically significant. The aggregate of correct responses to the 42 tested questions was used to compute each respondent's overall KAP score for food safety. Each accurate response received one point, while each incorrect answer received zero points. The total KAP scores of respondents were separated into two groups. Respondents who scored more than or equal to 60% of the overall KAP score were thought to have a high level of knowledge, positive attitudes, and appropriate practices. In comparison, less than 60% would be considered inadequate knowledge, negative attitudes, and inappropriate practices. The 60% passing rate (cut-off value) was determined by research done by Ayaz, Priyadarshini, and Jaiswal. [28].

3. Results and discussion:

3.1. Respondents characteristics

In all, 1126 Jordanians from various regions took part in the study. The respondents aged 24 years or younger represented 18.5% of the total, the respondents aged 25–44 years comprised 55.2% of the total, and the respondents aged 45 years or older comprised 26.6% of the total (Fig 1). The majority of the respondents were married (68.1%), and 31.9% were single. Approximately 15% of those polled had a higher university education degree (MSc or Ph.D.), 13.2% had a Diploma degree, 14% were uneducated or had only a 12-year school education, and 57.1% had a

Bachelor degree. A third (33.3%) of those polled had fewer than 5 years of experience in preparing food, 29.9% had between 5 and 10 years, and 36.8% had more than 10 years. Approximately 72% of respondents said the COVID-19 epidemic raised their concerns about food safety.

Fig 1: Characteristics of women food handlers at homes in Jordan (n=1126)

3.2. Food safety KAP aspects

3.2.1. Total food safety KAP.

The overall food safety KAP score was computed by summing the right responses to the survey's 42 questions. The minimum total KAP score for all examined factors of food safety was 10, and the maximum was 38 out of 42. This study offers information concerning food safety KAP of women food handlers in households in Jordan during the COVID-19 pandemic. Because of the large sample size (1126), the results could effectively reflect the status of food safety KAP. The mean total food safety KAP score was 22.1/42, corresponding to 52.6% of the questions answered correctly (Table 1). According to the findings of this study, women food handlers in Jordan had inadequate knowledge, negative attitudes, and inappropriate practices (an overall food safety KAP score of ≤ 60%). The percentage of total correct answers in the current study (52.6 %) was lower than that of women in the Sharjah-United Arab Emirate (57.4 %) [29]. The current study's findings on food safety KAP are similar to those of [9, 30, 31, 32, 33]. However, the findings of this study disagree with that reported by Okour et al., in which most consumers in Jordan exhibited good food safety knowledge [13]. This discrepancy in results between the current study and Okour et al. study might be due to differences in the populations studied in both investigations, with only females participating in the current study and both females and males participating in Okour et al. study. Furthermore, Okour et al. stated that one of their study's limitations was that the survey

questions used in the study did not cover all aspects of food safety knowledge. In contrast, the current study covered almost all aspects of food safety knowledge, attitudes, practices, and COVID-19.

Table 1: Food safety KAP score (mean and percentage) of respondents

Aspect	Score Mean ± Std. Error	Score percent[1] Mean ± Std. Error
Total food safety KAP (42 questions)	22.1±0.1	52.6±0.3
Personal Hygiene (9 questions)	7.2±0.03	79.9±0.4
Cross-contamination prevention (5 questions)	1.4±0.03	28.1±0.6
Cleaning and sanitation (4 questions)	2.6±0.03	64.7±0.6
Safe storage, thawing, cooking, keeping, and reheating of foods (12 questions)	5.1±0.06	42.8±0.5
Health issues that would affect food safety (3 questions)	1.1±0.02	37.9±0.8
Symptoms of foodborne illnesses (5 questions)	2.6±0.03	51.3±0.7
COVID-19 (4 questions)	2.1±0.03	51.8±0.7

[1] Score percent = (Score/maximum*100)

3.2.2. "Personal hygiene" aspect

Correct answers to the "personal hygiene" aspect are presented in Fig 2. They had the highest mean knowledge and practice ratings in personal hygiene, highlighting that women food handlers at homes in Jordan had good knowledge and proper practices about personal hygiene. This is analogous to what Farahat, El-Shafie, and Waly have previously discovered among Saudi women [34]. Hand washing was prevalent, as predicted. More than 90% of respondents could identify six situations when handwashing is required, and 38.5% said they wash their hands after using mobile phones. A study conducted by Osaili, Saeed, Taha, Omar Adrees, & Hasan among females living

in Dubai reported that 57.9% of them knew that hand washing was not necessary after every stage of food preparation [35]. Moreover, 64.7% of the respondents mentioned knowing the correct way to wash their hands. This is comparable to that reported by Ayaz et al., in which they stated that 65.3% of Saudi mothers knew the correct way of handwashing [28]. Also, the current study stated that 38.9% of the respondents knew that the duration of handwashing is 20 sec or more. To avoid foodborne infections, the Centers for Disease Control and Prevention (CDC) recommend washing hands with hot water and detergent for at least 20 seconds. [36]. Other researchers have proved that food handlers had a high level of personal hygiene knowledge and practice [37, 38, 39].

Fig 2: The proportion of accurate responses for the "personal hygiene" element was given by 1126 respondents.

3.2.3. "Cross-contamination prevention" aspect

The present study found that cross-contamination prevention had the most unfavorable attitudes and incorrect practices, with an average score of 28.1%. Less than half (45%) of those polled said they cover their hair when handling food (Fig 3). Barely 15.5 and 3.6% of respondents said they use gloves and masks when handling food, respectively. In terms of wearing gloves, a similar conclusion was reached among food handlers in Egypt's Sohag Governorate (18.6%) [40]. However, 35.1% of respondents correctly responded to a question on preventing food cross-contamination.

Fig 3: The proportion of accurate responses for the "cross-contamination prevention" element was given by 1126 respondents.

Nevertheless, a higher percentage was observed in a study conducted among Canadians [41]. Furthermore, in the current survey, just 41.3 % segregate ready-to-eat food from raw food in the refrigerator. In contrast, the majority of Sharjah's women (70.1%) do so [29]. Redmond and

Griffith stated that poor food-handling practices are indeed widespread in homes during food preparation [42].

3.2.4. “Cleaning and sanitation” aspect

Cleaning and sanitation knowledge and practices were highly rated, with an average score of 64.7%. The proportion of women who knew how to wipe and sanitize the kitchen countertop (85.4%) was greater than the proportion of Slovenian customers (59.7%) [32]. Given that the kitchen sink should always be disinfected on a daily basis to prevent foodborne diseases [43], About 87 % of respondents in the present survey clean their kitchen sink on a regular basis, as shown in Fig 4. This conclusion was remarkably identical to what women in Lahore reported (82.1%) [44]. Conversely, 95.9% of Lahore women were aware that washing vegetables and fruits in running cold water made them safe to consume [44], while in the current study, only 55.5% knew that. Finally, just 31.1% said soaking and washing the kitchen sponge in water was the least secure method of disinfection. Multiple studies have found kitchen sponges to be an important reservoir of food germs that can lead to contamination [35, 45, 46].

Fig 4: The proportion of accurate responses for the "cleaning and sanitation" element was given by 1126 respondents.

3.2.5. “Safe storage, thawing, cooking, keeping and reheating of foods” aspect

A Low KAP score was obtained among women in our study about food temperatures, with a mean score of 42.8%, which is similar to that reported in Australia [47] and Ireland [48]. According to the WHO, 45.6% of foodborne illness outbreaks owed by temperature abuse during food preparation [49]. The most correct answers (87.6%) were about purchasing frozen food at the end of a grocery run and never refreezing defrosted frozen meat, poultry, or fish for later use (Fig

5). These findings are better than that reported by Jevšnik et al. [32], in which only 9.9 and 70% of the Slovenian consumers knew that frozen food must be purchased at the end of shopping, and defrosted raw meat should not be refrozen for later use. A substantial majority of the right answers (70.9%) were about storing leftovers in the fridge for no more than two days. However, just 31.1% of household women heat leftovers until they are boiling, and 54.7% quickly discard unfinished leftovers. The United States Department of Agriculture (USDA) stated that leftovers should be handled according to hygienic procedures, placed in clean dishes, cooled immediately, sealed, and refrigerated at 4–7 °C for no more than three days, or frozen if stored for a longer period of time. However, women in Lahore had a higher rate (90.7%) [44]. Shallow knowledge was observed regarding checking whether poultry was sufficiently cooked by its temperature (4.7%), which is similar to that reported among women in Lahore (0%) [44]. Also, negative attitudes were noticed regarding food keeping temperatures, in which 39.9, 5.8, 32.6, and 40.1% thought refrigerated ready-to-eat food should indeed be stored at 1-4°C, heated ready-to-eat food at >60°C, frozen food at -18°C, and chicken tissue at 74°C during cooking. Similarly, research done in Jordan's north found that 34, 21, and 33% of female university students, respectively, knew the correct food-keeping temperatures in the refrigerator, the freezer, and the food's internal temperature during cooking [51]. Lastly, only 23.3% of women stated that precooked meals that would be served three hours later should be refrigerated and then warmed when ready to eat.

Fig 5: The proportion of accurate responses for the "safe storage, thawing, cooking, keeping, and reheating of foods" element was given by 1126 respondents.

3.2.6. "Health issues that would affect food safety" aspect

With a score percent mean of 37.9%, respondents in our survey had inadequate knowledge and unfavorable attitudes about "health issues that would affect food safety." Pathogenic

microorganism-contaminated meals do not have to taste, smell, or even appear different from safe-to-eat foods [43], but only 18.2% knew that (Fig 6). In our study, over two-thirds of the women (63.5 %) were aware that handling foods with an open cut on the back of their hands was hazardous. This finding indicates that food handlers in Jordan were more aware of this issue than those in analogous studies done in China, where just 25.5% of respondents were aware of the right response [31], 29.1% in Brazil [52], and 19.6% in Greece [53]. Lastly, 32.1% said that tasting meals with the same utensils used to mix them is the riskiest way to do so while cooking.

Fig 6: The proportion of accurate responses for the "health issues that would affect food safety" element was given by 1126 respondents.

3.2.7. "Symptoms of foodborne illnesses" aspect

With a mean score of 51.3 percent, the subjects of this study demonstrated a poor awareness of foodborne microorganisms and illnesses. Only 26, 24.6, and 37.5 percent of those polled were aware that hypertension, low blood sugar, and a cold or cough, respectively, are not indicators of foodborne disease (Fig 7). However, the vast majority of them (98.8%) identified diarrhea and vomiting as symptoms of foodborne disease, which is not surprising given that the most widely mentioned symptoms of foodborne gastroenteritis in the news are diarrhea and vomiting. Finally, 69.4 percent of those polled said that headaches indicate food poisoning. In Portugal, however, 85 percent of those surveyed were able to recognize the signs and symptoms of food poisoning [54].

Fig 7: The proportion of accurate responses for the "symptoms of foodborne illnesses" element was given by 1126 respondents.

3.2.8. "COVID-19 KAP" Aspect

Despite the fact that 68.4% of subjects disinfected food containers well before consuming or storing them, they had inadequate understanding and negative attitudes toward COVID-19 (Fig 8).

Only 46.4 and 25.4% of subjects were aware that the COVID-19 virus cannot replicate or be transmitted through food, respectively. About two-thirds (67.1%) of subjects thought the COVID-19 virus could persist on rough surfaces for a day (or days). A study conducted by Van Doremalen et al. reported that COVID-19 could persist on inanimate surfaces for 48 to 72 h [55]. Also, WHO concluded that COVID-19 could not proliferate or be transferred by food, but owing to the virus' persistence on surfaces, it may be a vector of virus transmission [56].

Fig 8: The proportion of accurate responses for the "COVID-19 KAP" element was given by 1126 respondents.

3.3. Improvements of subjects' food safety precautions during COVID-19

The perspectives of respondents about the COVID-19 epidemic and food safety are summarized in Table 2. Most participants (76.7%) were concerned about spreading the COVID-19 virus through food they provided to others, and 83% were concerned about spreading the infection while preparing meals. In a similar study, 90.1% of the participants among Brazilians indicated that they are more cautious regarding food hygiene practices and attitudes since they think food can transmit the COVID-19 viral infection [57]. As mentioned before, the COVID-19 epidemic heightened 71.8% of participants' awareness concerning food safety, and 13.3% said it did not. High percentages (86.3, 92.3, 69.4, and 91.8%) of the respondents revealed that the COVID-19 epidemic improved their food safety precautions by reducing contamination of food, maintaining high levels of human hygiene, managing food temperature, and sanitizing and cleaning food contact areas, correspondingly. Similarly, Thomas and Feng stated that the COVID-19 pandemic had affected food handlers' food safety practices in the United States. Hand hygiene, washing food packaging, and using a food thermometer are all examples of this [58]. Lastly, the

WHO mobilized people worldwide to increase adherence to hand hygiene to save lives during the COVID-19 pandemic [59].

Table 2: Query statements and responses on the improvements of subjects' food safety precautions during COVID-19

Statement	KAP	Category	Frequency	Percent %
• Worrying about transmitting COVID-19 virus infection through the food shared with others	Attitude	Yes<br>Neutral<br>No	864<br>143<br>119	76.7<br>12.7<br>10.6
• Worrying about transmitting the COVID-19 viral infection to others while preparing food while infected with it	Attitude	Yes<br>Neutral<br>No	935<br>87<br>104	83.0<br>7.7<br>9.2
• Increasing food safety concerns during the COVID-19 epidemic	Attitude	Yes<br>Neutral<br>No	808<br>168<br>150	71.8<br>14.9<br>13.3
• Enhancing food safety measures in decreasing food contamination during the COVID-19 epidemic	Attitude	Yes<br>Neutral<br>No	972<br>100<br>54	86.3<br>8.9<br>4.8
• Enhancing food safety measures in maintaining personal hygiene at a high level during the COVID-19 epidemic	Attitude	Yes<br>Neutral<br>No	1039<br>49<br>38	92.3<br>4.4<br>3.4
• Enhancing food safety measures in controlling the temperature of food during storage, cooking, keeping, and reheating during the COVID-19 epidemic	Attitude	Yes<br>Neutral<br>No	782<br>229<br>115	69.4<br>20.3<br>10.2
• Enhancing food safety measures in cleaning and sanitation of food contact surfaces during the COVID-19 epidemic	Attitude	Yes<br>Neutral<br>No	1034<br>57<br>35	91.8<br>5.1<br>3.1

3.4. The relationship between food safety KAP scores and overall respondent characteristics

Social determinants play an essential role in determining consumers' food safety KAP. No significant relationship was observed between marital status and overall food safety KAP (Table

3). Furthermore, significant associations were observed between respondents' overall food safety KAP scores and age, educational level, experience, and the effect of the COVID-19 epidemic on food safety concerns (*P < 0.05*). The results showed that respondents had significantly higher food safety KAP scores if they were more than 44 years old (22.5%) compared to respondents who were less than 24 years of age (21.6%). Also, respondents who had higher university education degree (MSc or Ph.D.) (23.07%) had significantly better food safety KAP scores than those who had a bachelor degree (22.07%) and uneducated or a 12-year school education (21.25%). Respondents had significantly better food safety KAP scores with more than ten experience years (22.7%) and within 5-10 experience years (22.3%) compared to respondents with less than five experience years (21.3%). Lastly, respondents who said the COVID-19 pandemic increased their concern about food safety (22.2%) and did not (22.4%) had received significantly higher KAP ratings for food safety than individuals who were neutral about that (21.5%). Similarly, there was a significant relationship between total food safety attitudes with each gender, age, educational level, and the degree of attention paid to the COVID-19 epidemic situation in a study conducted in China [21]. Previous studies have demonstrated significant differences between food safety KAP and demographics, such as gender, age, race, and income [60]. Also, Langiano et al. reported significant differences in gender, marital status, educational level, and occupation [9].

Table 3: The relationship between food safety KAP scores and overall respondent characteristics

Respondents characteristics		Score Mean ± Std. Error	Score percent[1] Mean ± Std. Error	P-value
Age[2]				
	≤24	$21.6^{b}\pm0.3$	51.5±0.8	
	25-44	$22.0^{ab}\pm0.2$	52.5±0.4	**0.048**
	≥45	$22.5^{a}\pm0.2$	53.7±0.6	
Educational Level[2]				
	Uneducated or a 12-year school education	$21.25^{b}\pm0.3$	50.6±0.8	**0.001**

	Diploma degree	$21.95^{ab}\pm0.3$	52.3 ± 0.7	
	Bachelor degree	$22.07^{b}\pm0.2$	52.6 ± 0.4	
	Higher university education (MSc or Ph.D.)	$23.07^{a}\pm0.4$	54.9 ± 0.8	
Marital Status[2]				
	Single	21.8 ± 0.2	51.8 ± 0.6	0.06
	Married	22.3 ± 0.1	53.0 ± 0.3	
Experience[2]				
	<5	$21.3^{b}\pm0.2$	50.7 ± 0.5	
	5-10	$22.3^{a}\pm0.2$	53.0 ± 0.5	**0.00**
	>10	$22.7^{a}\pm0.2$	54.1 ± 0.4	
Region[2]				
	North	$21.8^{b}\pm0.2$	51.9 ± 0.5	
	Central	$22.3^{a}\pm0.2$	53.2 ± 0.4	**0.03**
	South	$21.4^{b}\pm0.4$	50.8 ± 1.0	
Increased food safety concerns during COVID-19 [3]				
	Yes	$22.2^{a}\pm0.1$	52.7 ± 0.3	
	Neutral	$21.5^{b}\pm0.4$	51.2 ± 0.9	**0.03**
	No	$22.4^{a}\pm0.4$	53.4 ± 0.9	

[1] Score percent equal to (Score/maximum*100)

[2] Using multivariate general linear model (GLM).

[3] Using Kruskal-Wallis Test.

Note: At $p \leq 0.05$, different letters for means within the same categories differ significantly. All factors were adjusted for one another.

3.5. The impact of demographic variables on food safety KAP scores using logistic regression analysis

Scores were separated into groups; (1) scores more than or equal to the 60th percentile, (2) scores less than the 60th percentile. The 60th percentile cut-off point was (25/42). All variables were adjusted for each other. The food safety KAP score of the subjects was significantly ($P < 0.05$) affected by experience; respondents with less than five experience years and those with 5–10 experience years were 1.91 and 1.53 times more likely to have a significantly lower food safety KAP score (OR 1.91 and 1.53) than those with ten experience years (Table 4). These findings are supported by many studies [34, 54, 61, 62, 63]. As expected, the food safety KAP score of the

subjects was also significantly (*P < 0.05*) affected by educational level; respondents who had a diploma degree and uneducated or had school education (≤12 years) were 2.39 and 2.16 times more likely to have significantly lower food safety KAP score (OR 2.39 and 2.16) than those who had higher university education (MSc or Ph.D.). Similarly, a study conducted among students in East Malaysia used a binary logistic test to determine the impact of socio-demographic variables on food safety knowledge. It found that educational level was one of the predictors of food safety knowledge [10].

Table 4: Food safety KAP predictors using logistic regression analysis

Variable [1]		OR (CI_{95})	P-value
Age			
	≤24	0.995 (0.55,1.80)	0.998
	25-44	1.16 (0.81,1.66)	0.42
	≥45	Reference	
Educational Level			
	Uneducated or a 12-year school education	2.16 (1.22,3.84)	0.01*
	Diploma degree	2.39 (1.33,4.29)	0.00*
	Bachelor degree	1.32 (0.88,1.96)	0.18
	Higher university education (MSc or Ph.D.)	Reference	
Marital Status			
	Single	0.84 (0.57,1.25)	0.39
	Married	Reference	
Experience			
	<5	1.91 (1.24,2.94)	0.00*
	5-10	1.53 (1.05,2.22)	0.03*
	>10	Reference	
Region			
	North	0.89 (0.44,1.80)	0.74
	Central	0.67 (0.34,1.33)	0.25
	South	Reference	
Increased food safety concerns during COVID-19			
	Yes	1.05 (0.68,1.62)	0.82
	Neutral	Reference	
	No	0.78 (0.45,1.35)	0.38

[1] All variables were adjusted to each other.

* P-value ≤ 0.05 was considered statistically significant

3.6. The connection between major food safety aspect ratings and improvements in participants' food safety measures during COVID-19

No significant connections were observed between the "cross-contamination prevention" and "personal hygiene" aspects scores and the subjects' attitudes toward decreasing food contamination and maintaining good personal hygiene during the COVID-19 epidemic (Table 5). However, there were significant connections between the "cleaning and sanitation" aspect score and the subjects' attitudes toward cleaning and sanitation of food contact surfaces during the COVID-19 epidemic ($p < 0.05$). Subjects who thought their precautions for cleaning and sanitation of food contact surfaces were enhanced (2.6%) and those who did not think so (2.9%) had a significantly higher score than those who were neutral (2.2%). Also, significant connections were observed between the aspect score of "safe storage, thawing, cooking, keeping, and reheating of food" and the subjects' attitudes toward controlling the temperature of food during the COVID-19 epidemic ($P < 0.05$). The results showed that the subjects who thought that their precautions were enhanced during the COVID-19 epidemic (5.2%) had a significantly greater score than those who did not (4.7%). In Brazil, 72% desired to learn more about sanitizing food and cleaning food packaging and were more concerned about the safety and hygiene of food contact surfaces [57]. Moreover, the COVID-19 pandemic has led to considerable changes in food purchasing, food handling, and hygiene practices in Lebanon, Jordan, and Tunisia [64]. Therefore, it is vital to conduct continuous comprehensive education and training programs for domestic food handlers, especially women, to protect themselves and their families against foodborne illnesses.

Table 5: The connection between major food safety aspect ratings and improvements in participants' food safety measures during COVID-19

Aspect	COVID-19 statement	Score Mean ± Std. Error	Score percent[1] Mean ± Std. Error	P-value
Food contamination prevention	Enhancing food safety measures in decreasing food contamination during the COVID-19 epidemic			
	Yes	2.6±0.0	64.8±0.7	
	Neutral	2.5±0.1	62.3±2.2	0.73
	No	2.7±0.1	67.6±3.1	
Personal Hygiene	Enhancing food safety measures in maintaining high personal hygiene during the COVID-19 epidemic			
	Yes	7.2±0.03	79.9±0.4	
	Neutral	7.0±0.2	78.0±2.0	0.53
	No	7.3±0.2	81.6±1.8	
Cleaning and sanitation	Enhancing food safety measures in cleaning and sanitation of food contact surfaces during the COVID-19 epidemic			
	Yes	2.6^{a}±0.03	65.0±0.65	
	Neutral	2.2^{b}±0.14	55.7±3.43	**0.02**
	No	2.9^{a}±0.15	72.1±3.66	
Safe storage, thawing, cooking, keeping, and reheating of foods	Enhancing food safety measures in controlling the temperature of food during storage, cooking, keeping, and reheating during the COVID-19 epidemic			
	Yes	5.2^{a}±0.07	43.6±0.6	
	Neutral	5.1^{ab}±0.13	42.1±1.1	**0.01**
	No	4.7^{b}±0.20	39.0±1.7	

[1] Score percent equal to (Score/maximum*100)

Note: At $p \leq 0.05$, different letters for means within the same categories differ significantly. All factors were adjusted for one another.

4. Conclusion

The present study reveals that during the COVID-19 epidemic, women food handlers in Jordan had limited knowledge, unfavorable attitudes, and unsafe food handling practices. It is crucial to

engage the public in continuous education regarding proper food handling practices. Food safety efforts are often focused on food supply chains, with little attention paid to food handling and practices in the home. Therefore, domestic food handlers require comprehensive education and training to protect family members from foodborne illnesses.

5. Limitation

Limitations of the study are that this research examined self-reported food safety practices, which are prone to bias by respondents. Also, questions used in the demographics part might not cover all social determinants that could impact food safety KAP, such as income, number of family members, and occupation.

Acknowledgments

The authors would like to thank Jordan University of Science and Technology's deanship of research for financing this effort. Volunteers are thanked for their assistance in providing relevant information during the research.

Author contributions

Conceptualization: Anas A. Al-Nabulsi, Tareq M. Osaili, Amin N. Olaimat

Data curation: Tasneem M. Al-Jaberi, Sawsan Mutlaq

Formal analysis: Tasneem M. Al-Jaberi

Funding acquisition: Anas A. Al-Nabulsi, Tareq M. Osaili, Amin N. Olaimat

Investigation: Tasneem M. Al-Jaberi, Sawsan Mutlaq

Methodology: Anas A. Al-Nabulsi, Tareq M. Osaili

Project administration: Anas A. Al-Nabulsi, Tareq M. Osaili

Resources: Tasneem M. Al-Jaberi, Sawsan Mutlaq

Supervision: Anas A. Al-Nabulsi, Tareq M. Osaili

Validation: Anas A. Al-Nabulsi, Tareq M. Osaili, Amin N. Olaimat

Visualization: Tasneem M. Al-Jaberi

Writing – original draft: Tasneem M. Al-Jaberi

Writing – review & editing: Anas A. Al-Nabulsi, Tareq M. Osaili, Amin N. Olaimat

References

1. WHO. Estimating the burden of foodborne diseases [Internet]. World Health Organization. World Health Organization; 2015 [cited 2022Nov28]. Available from: https://www.who.int/activities/estimating-the-burden-of-foodborne-diseases
2. Gargouri N, Walke H, Belbeisi A, Hadadin A, Salah S, Ellis A, et al. Estimated burden of human Salmonella, Shigella, and Brucella infections in Jordan, 2003-2004. Foodborne Pathogens and Disease [Internet]. 2009;6(4):481–6. Available from: http://dx.doi.org/10.1089/fpd.2008.0192
3. News: More than 800 hospitalized in Jordan food poisoning outbreak. [Internet]. Home Page - minister of health. Ministry of Health: Hashemite Kingdom of Jordan; 2020 [cited 2022Jan]. Available from: https://www.moh.gov.jo/DetailsPage/MOH_AR/NewsDetailsAR.aspx?ID=643
4. WHO. Food safety: What you should know [Internet]. World Health Organization. World Health Organization; 2015 [cited 2022Nov28]. Available from: https://apps.who.int/iris/handle/10665/160165
5. Desta M, Asrat D, Woldeamanuel Y, Nigusie D. Prevalence of intestinal parasites and Salmonella and Shigella among food handlers at food service establishments in the main campus and Health Sciences College of Hawassa University, Hawassa, Ethiopia. The Ethiopian Journal of Health Development. 2014;28(1):PDF-.
6. Odeyemi OA, Sani NA, Obadina AO, Saba CK, Bamidele FA, Abughoush M, et al. Food Safety Knowledge, attitudes and practices among consumers in developing countries: An international survey. Food Research International. 2019;116:1386–90.

7. European Food Safety Authority, European Centre for Disease Prevention and Control. The European Union one health 2019 zoonoses report. EFSA Journal. 2021 Feb;19(2):e06406.
8. Unusan N. Consumer Food Safety Knowledge and practices in the home in Turkey. Food Control. 2007;18(1):45–51.
9. Langiano E, Ferrara M, Lanni L, Viscardi V, Abbatecola AM, De Vito E. Food safety at home: knowledge and practices of consumers. Journal of Public Health [Internet]. 2012;20(1):47–57. Available from: http://dx.doi.org/10.1007/s10389-011-0437-z
10. Ruby GE, Ungku Zainal Abidin UF, Lihan S, Jambari NN, Radu S. A cross sectional study on food safety knowledge among adult consumers. Food Control. 2019;99:98–105.
11. Wu Y-ning, Liu X-mei, Chen Q, Liu H, Dai Y, Zhou Y-jing, et al. Surveillance for Foodborne Disease Outbreaks in China, 2003 to 2008. Food Control. 2018;84:382–8.
12. Epidemiological situation: Health indicators [Internet]. Home Page - minister of health. Ministry of Health: Hashemite Kingdom of Jordan; 2018 [cited 2022Jan]. Available from: https://www.moh.gov.jo/Pages/viewpage.aspx?%20pageID=240
13. Okour AM, Alzein E, Saadeh R, alfaqih M. Food Safety Knowledge among Jordanians: A national study. Food Control. 2020;114:107216.
14. M, Galanakis IM, Aldawoud TMS, Galanakis CM. Safety of foods, food supply chain and environment within the COVID-19 pandemic. Trends in Food Science & Technology. 2020;102:293–9.
15. WHO. Coronavirus disease (covid-19): How is it transmitted? [Internet]. World Health Organization. World Health Organization; 2020 [cited 2022Dec2]. Available from:

https://www.who.int/news-room/questions-and-answers/item/coronavirus-disease-covid-19-how-is-it-transmitted

16. WHO. Coronavirus disease (COVID-19) [Internet]. World Health Organization. World Health Organization; 2020 [cited 2022Dec2]. Available from: https://www.who.int/health-topics/coronavirus#tab=tab_1
17. FAO. COVID-19: Guidance for preventing transmission of COVID-19 within food businesses [Internet]. Fao.org. 2021 [cited 2022 Nov 25]. Available from: https://www.fao.org/documents/card/ar/c/cb6030en/
18. Omar SS. Impact of pandemic crisis: COVID-19 on food safety knowledge, attitudes and practices among food workers in Jordan. EurAsian Journal of Biosciences. 2020;14(2):3581–6.
19. Hatabu A, Mao X, Zhou Y, Kawashita N, Wen Z, Ueda M, et al. Knowledge, attitudes, and practices toward COVID-19 among university students in Japan and associated factors: An online cross-sectional survey. PLoS One [Internet]. 2020;15(12):e0244350. Available from: http://dx.doi.org/10.1371/journal.pone.0244350
20. Mayurnikova L, Koksharov A, Krapiva T. Food safety practices in catering during the coronavirus COVID-19 pandemic. Foods and Raw materials [Internet]. 2020;8(2):197–203. Available from: http://dx.doi.org/10.21603/2308-4057-2020-2-197-203
21. Luo Y, Chen L, Xu F, Gao X, Han D, Na L. Investigation on knowledge, attitudes and practices about food safety and nutrition in the China during the epidemic of Corona virus disease 2019. Public Health Nutrition. 2020;24(2):267–74.
22. Ranaei V, Pilevar Z, Hosseini H. Food safety practices in covid-19 pandemic. Journal of Food Quality and Hazards Control. 2020.

23. Omar S. Hygienic Food Practice among Food Workers in Jordanian Food Factories during pandemic crisis: COVID-19: comparative study. Research Square. 2020.

24. Philimis AD, Sancheti S. Familial Bonding Owing to COVID-19 Pre and Post Lockdown. In: New Normal;reinventing professional life and familial bonding in the post covid 19 ERA. S.l.: BLOOMSBURY INDIA; 2021.

25. Estimated Population of the Kingdom by Governorate, Locality Sex and Households,2020 [Internet]. Department of Statistics. 2020 [cited 2022Nov21]. Available from: https://jordankmportal.com/resources/estimated-population-of-the-kingdom-by-governorate-locality-sex-and-households-2017

26. Limon MR. Food safety practices of food handlers at home engaged in online food businesses during COVID-19 pandemic in the Philippines. Curr Res Food Sci [Internet]. 2021;4:63–73. Available from: http://dx.doi.org/10.1016/j.crfs.2021.01.001

27. Osaili TM, Abu Jamous DO, Obeidat BA, Bawadi HA, Tayyem RF, Subih HS. Food safety knowledge among food workers in restaurants in Jordan. Food Control. 2013;31(1):145–50.

28. Ayaz WO, Priyadarshini A, Jaiswal AK. Food safety knowledge and practices among Saudi mothers. Foods. 2018 Nov 25;7(12):193.

29. Saeed BQ, Osaili TM, Taha S. Foodborne diseases risk factors associated with food safety knowledge and practices of women in Sharjah-united arab emirate. Food Control. 2021;125:108024.

30. Ali IE. *Food Safety Knowledge and Practices among Women in Nile river state* (Doctoral dissertation, Sudan Uiversity of Cince and Technologe). 2015.

31. Gong S, Wang X, Yang Y, Bai L. Knowledge of food safety and handling in households: A survey of food handlers in Mainland China. Food Control [Internet]. 2016;64:45–53. Available from: http://dx.doi.org/10.1016/j.foodcont.2015.12.006
32. Jevšnik M, Hlebec V, Raspor P. Consumers' awareness of food safety from shopping to eating. Food Control [Internet]. 2008;19(8):737–45. Available from: http://dx.doi.org/10.1016/j.foodcont.2007.07.017
33. Tomaszewska M, Trafialek J, Suebpongsang P, Kolanowski W. Food Hygiene Knowledge and practice of consumers in Poland and in Thailand - A survey. Food Control. 2018;85:76–84.
34. Farahat MF, El-Shafie MM, Waly MI. Food safety knowledge and practices among Saudi women. Food Control [Internet]. 2015;47:427–35. Available from: http://dx.doi.org/10.1016/j.foodcont.2014.07.045
35. Osaili TM, Saeed BQ, Taha S, Omar Adrees A, Hasan F. Knowledge, practices, and risk perception associated with foodborne illnesses among females living in Dubai, United Arab Emirates. Foods. 2022;11(3):290.
36. CDC. When and how to wash your hands [Internet]. Centers for Disease Control and Prevention. Centers for Disease Control and Prevention; 2022 [cited 2022Nov21]. Available from: https://www.cdc.gov/handwashing/when-how-handwashing.html
37. Fawzi M, Shama ME. Food safety knowledge and practices among women working in Alexandria University, Egypt. J Egypt Public Health Assoc. 2009;84(1–2):95–117.
38. Lum A, Albrecht J, Yaseen M, Litchfield R, Ritter-Gooder P. Food-handling Practices and Knowledge among Families with Young Children. FOOD PROTECTION TRENDS

[Internet]. 2013 [cited 2022Nov25];33(6). Available from: https://www.foodprotection.org/files/food-protection-trends/Nov-Dec-13-Albrecht.pdf

39. Patil SR, Cates S, Morales R. Consumer Food Safety Knowledge, practices, and demographic differences: Findings from a meta-analysis. Journal of Food Protection. 2005;68(9):1884–94.

40. Hamed A, Mohammed N. Food safety knowledge, attitudes and self-reported practices among food handlers in Sohag Governorate, Egypt. Eastern Mediterranean Health Journal [Internet]. 2020;26(4):374–81. Available from: http://dx.doi.org/10.26719/emhj.19.047

41. Nesbitt A, Majowicz S, Finley R, Marshall B, Pollari F, Sargeant J, et al. High-risk food consumption and food safety practices in a Canadian community. Journal of Food Protection. 2009;72(12):2575–86.

42. Redmond EC, Griffith CJ. Consumer Food Handling in the home: A Review of Food Safety Studies. Journal of Food Protection. 2003;66(1):130–61.

43. CDC. Foods that can cause food poisoning [Internet]. Centers for Disease Control and Prevention. Centers for Disease Control and Prevention; 2022 [cited 2022Nov21]. Available from: https://www.cdc.gov/foodsafety/foods-linked-illness.html

44. Naeem N, Raza S, Mubeen H, Siddiqui SA, Khokhar R. Food Safety Knowledge, attitude, and food handling practices of household women in Lahore. Journal of Food Safety. 2018;38(5).

45. Osaili T, Shaker Obaid R, Taha S, Kayyaal S, Ali R, Osama M, et al. A cross-sectional study on food safety knowledge amongst domestic workers in the UAE. British Food Journal. 2021;124(3):1009–21.

46. Sharma M, Eastridge J, Mudd C. Effective household disinfection methods of kitchen sponges. Food Control. 2009;20(3):310–3.
47. Jay LS, Comar D, Govenlock LD. A national Australian food safety telephone survey. Journal of Food Protection [Internet]. 1999;62(8):921–8. Available from: http://dx.doi.org/10.4315/0362-028x-62.8.921
48. Kennedy J, Jackson V, Blair IS, McDowell DA, Cowan C, Bolton DJ. Food safety knowledge of consumers and the microbiological and temperature status of their refrigerators. Journal of Food Protection [Internet]. 2005;68(7):1421–30. Available from: http://dx.doi.org/10.4315/0362-028x-68.7.1421
49. WHO. Foodborne disease outbreaks: Guidelines for investigation and Control [Internet]. World Health Organization. World Health Organization; 2008 [cited 2022Nov28]. Available from: https://www.who.int/publications-detail-redirect/9789241547222
50. USDA. Food Safety and Inspection Service [Internet]. Leftovers and Food Safety | Food Safety and Inspection Service. USDA; 2020 [cited 2022Nov26]. Available from: https://www.fsis.usda.gov/food-safety/safe-food-handling-and-preparation/food-safety-basics/leftovers-and-food-safety
51. Osaili TM, Obeidat BA, Abu Jamous DO, Bawadi HA. Food Safety Knowledge and practices among college female students in north of Jordan. Food Control. 2011;22(2):269–76.
52. Uggioni PL, Salay E. Consumer knowledge concerning safe handling practices to prevent microbiological contamination in commercial restaurants and socio-demographic characteristics, Campinas/SP/Brazil. Food Control. 2012;26(2):331–6.

53. Lazou T, Georgiadis M, Pentieva K, McKevitt A, Iossifidou E. Food safety knowledge and food-handling practices of Greek university students: A questionnaire-based survey. Food Control [Internet]. 2012;28(2):400–11. Available from: http://dx.doi.org/10.1016/j.foodcont.2012.05.027
54. Carbas B, Cardoso L, Coelho AC. Investigation on the knowledge associated with foodborne diseases in consumers of northeastern Portugal. Food Control. 2013 Mar 1;30(1):54-7.
55. van Doremalen N, Bushmaker T, Morris DH, Holbrook MG, Gamble A, Williamson BN, et al. Aerosol and surface stability of SARS-COV-2 as compared with SARS-COV-1. New England Journal of Medicine. 2020;382(16):1564–7.
56. WHO. COVID-19 and food safety: guidance for food businesses: interim guidance, April 07, 2020 [Internet]. World Health Organization. World Health Organization and Food and Agriculture Organization of the United Nations; 2020 [cited 2022Dec2]. Available from: https://apps.who.int/iris/bitstream/handle/10665/331705/WHO-2019-nCoV-Food_Safety-2020.1-eng.pdf?sequence=1
57. Ferreira Rodrigues J, Cunha dos Santos Filho MT, Aparecida de Oliveira LE, Brandemburg Siman I, Barcelos Ade, de Paiva Anciens Ramos GL, et al. Effect of the COVID-19 pandemic on food habits and perceptions: A study with Brazilians. Trends in Food Science & Technology. 2021;116:992–1001.
58. Thomas MS, Feng Y. Food handling practices in the era of covid-19: A mixed-method longitudinal needs assessment of consumers in the United States. Journal of Food Protection. 2021;84(7):1176–87.

59. WHO. WHO Save lives: Clean your hands - in the context of covid-19 [Internet]. World Health Organization. World Health Organization; 2020 [cited 2022Dec2]. Available from: https://www.who.int/publications/m/item/save-lives-clean-your-hands-in-the-context-of-covid-19

60. Al-Sakkaf A. Campylobacteriosis in New Zealand: a new twist to the tale? Part two (the consumer and the regulator). Food control. 2013 Oct 1;33(2):562-6.

61. Brannon LA, York VK, Roberts KR, Shanklin CW, Howells AD. Appreciation of food safety practices based on level of experience. Journal of Foodservice Business Research. 2009 Jun 5;12(2):134-54.

62. Sharafie K, Safari Y, Karimaei M, Asadi F, Ghayebzadeh M, Motlagh ZJ, et al. The role of educational intervention in changing knowledge and attitudes of rural homemakers in relation to Food Safety and hygiene: A case study: Iran (2016). Annals of Tropical Medicine and Public Health. 2017;10(4):1024.

63. Shori AB. Awareness and knowledge about food spoilage and principles of food preservation among Saudi women in Jeddah. Journal of Food: Microbiology, Safety & Hygiene. 2017;02(02).

64. Faour-Klingbeil D, Osaili TM, Al-Nabulsi AA, Jemni M, Todd ECD. An on-line survey of the behavioral changes in Lebanon, Jordan and Tunisia during the COVID-19 pandemic related to food shopping, food handling, and hygienic practices. Food Control [Internet]. 2021;125(107934):107934. Available from: http://dx.doi.org/10.1016/j.foodcont.2021.107934

Figure
Click here to access/download;Figure;Food safety KAP among households figures 30.12.2022.docx

1 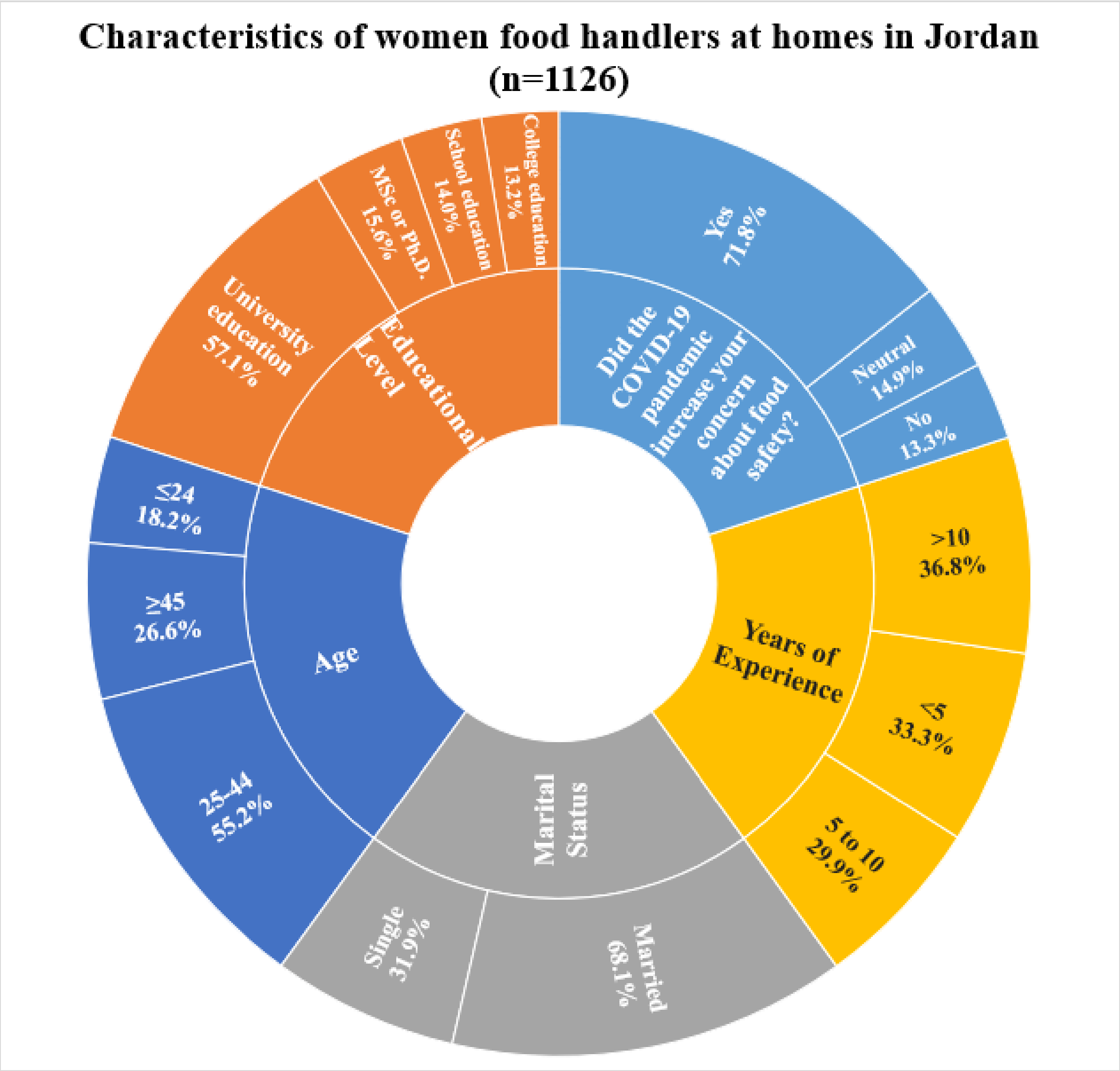

2 **Fig 1**

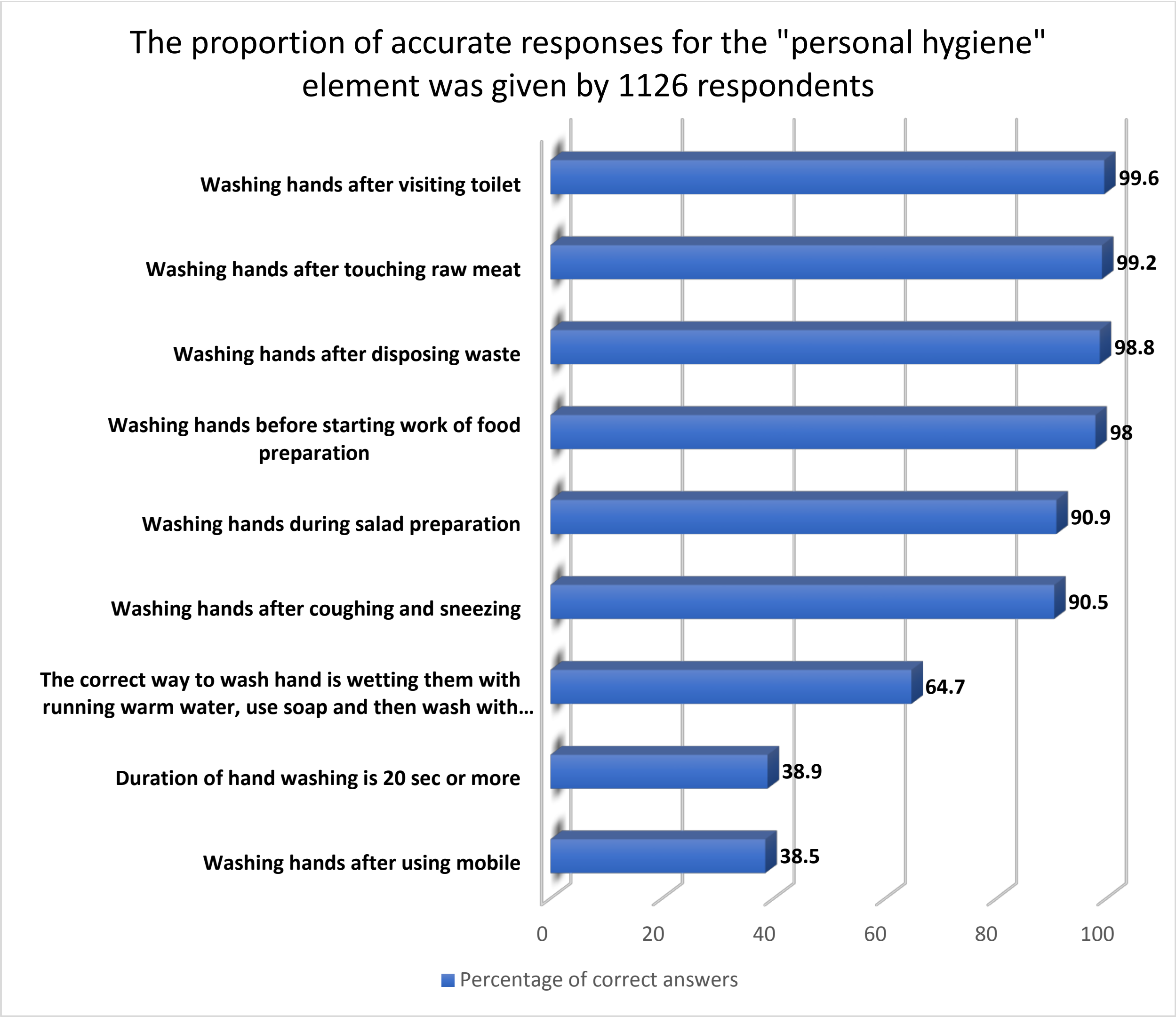
The proportion of accurate responses for the "personal hygiene" element was given by 1126 respondents
Washing hands after visiting toilet
Washing hands after touching raw meat
Washing hands after disposing waste
Washing hands before starting work of food preparation
Washing hands during salad preparation
Washing hands after coughing and sneezing
The correct way to wash hand is wetting them with running warm water, use soap and then wash with…
Duration of hand washing is 20 sec or more
Washing hands after using mobile
99.6
99.2
98.8
98
90.9
90.5
64.7
38.9
38.5
0
20
40
60
80
100
Percentage of correct answers

Fig 2

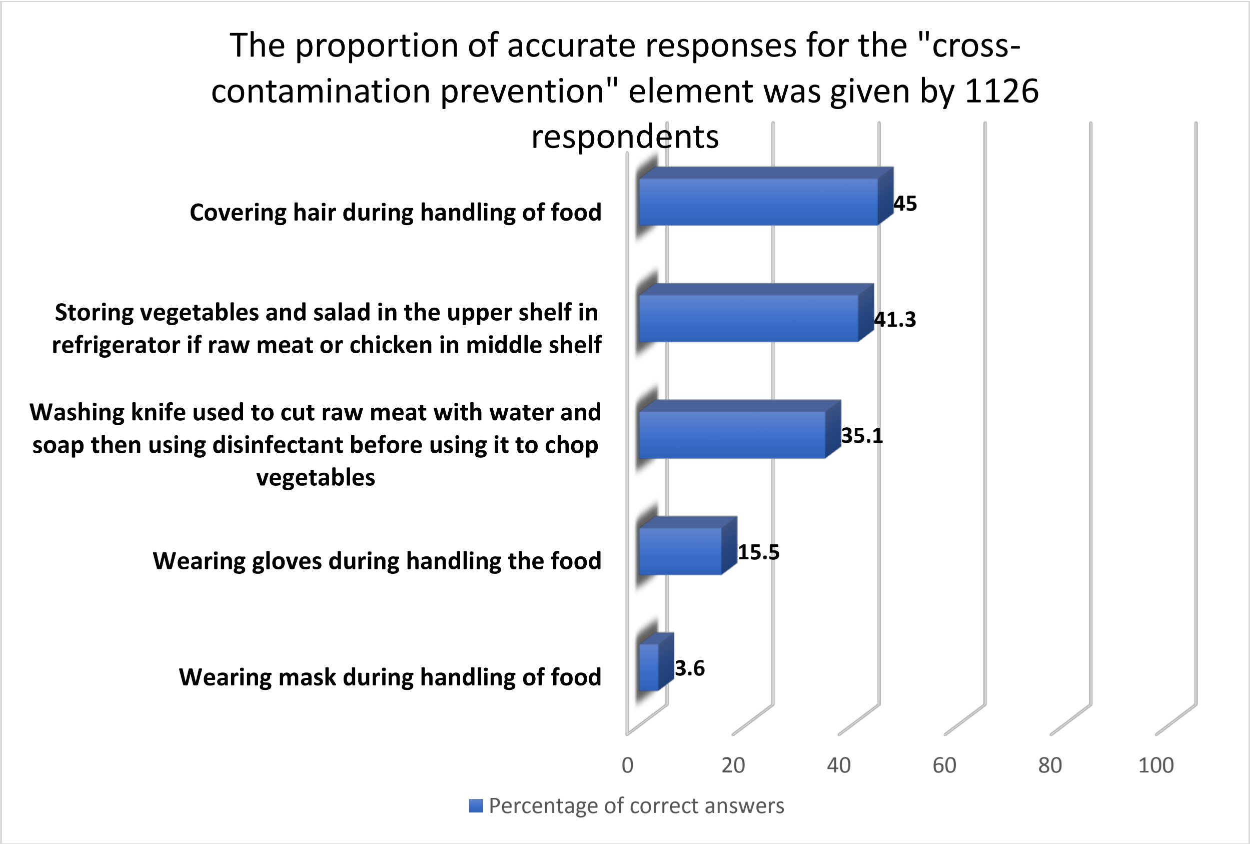
The proportion of accurate responses for the "cross-contamination prevention" element was given by 1126 respondents
Covering hair during handling of food
Storing vegetables and salad in the upper shelf in refrigerator if raw meat or chicken in middle shelf
Washing knife used to cut raw meat with water and soap then using disinfectant before using it to chop vegetables
Wearing gloves during handling the food
Wearing mask during handling of food
45
41.3
35.1
15.5
3.6
0
20
40
60
80
100
Percentage of correct answers

Fig 3

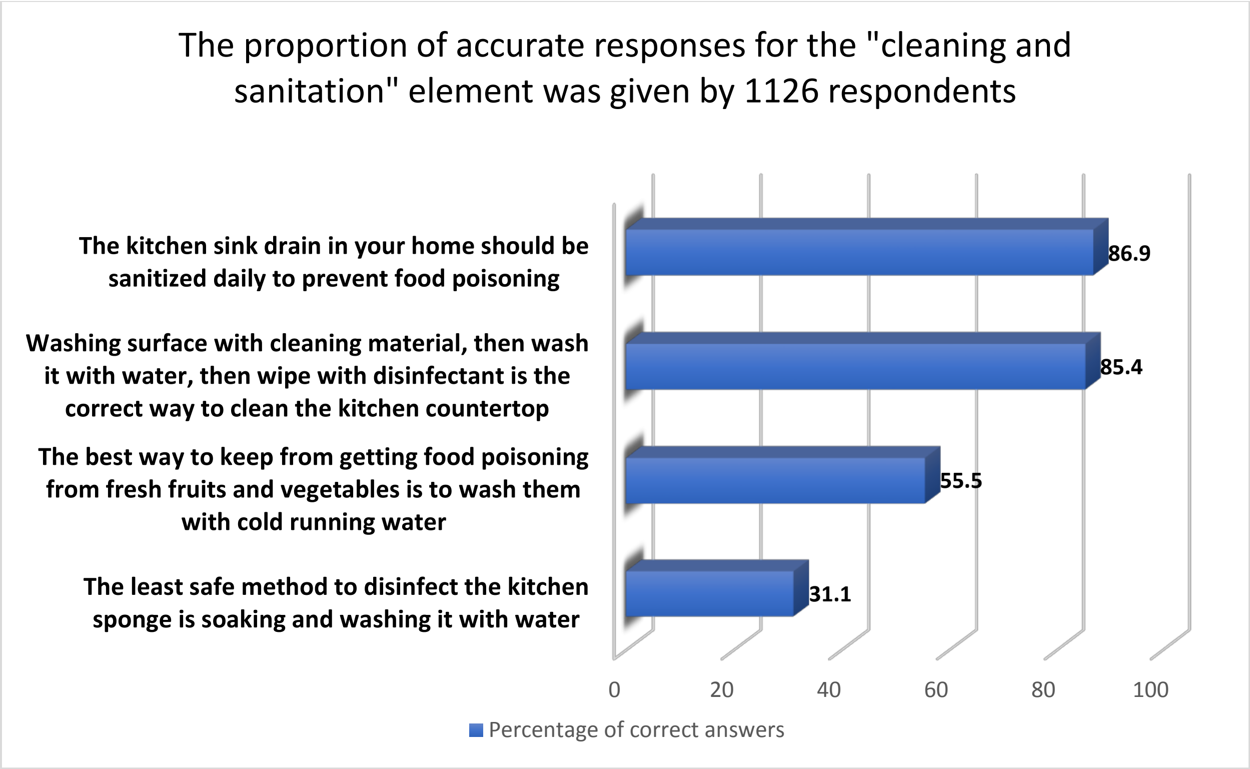
The proportion of accurate responses for the "cleaning and sanitation" element was given by 1126 respondents
The kitchen sink drain in your home should be sanitized daily to prevent food poisoning
Washing surface with cleaning material, then wash it with water, then wipe with disinfectant is the correct way to clean the kitchen countertop
The best way to keep from getting food poisoning from fresh fruits and vegetables is to wash them with cold running water
The least safe method to disinfect the kitchen sponge is soaking and washing it with water
86.9
85.4
55.5
31.1
0
20
40
60
80
100
Percentage of correct answers

Fig 4

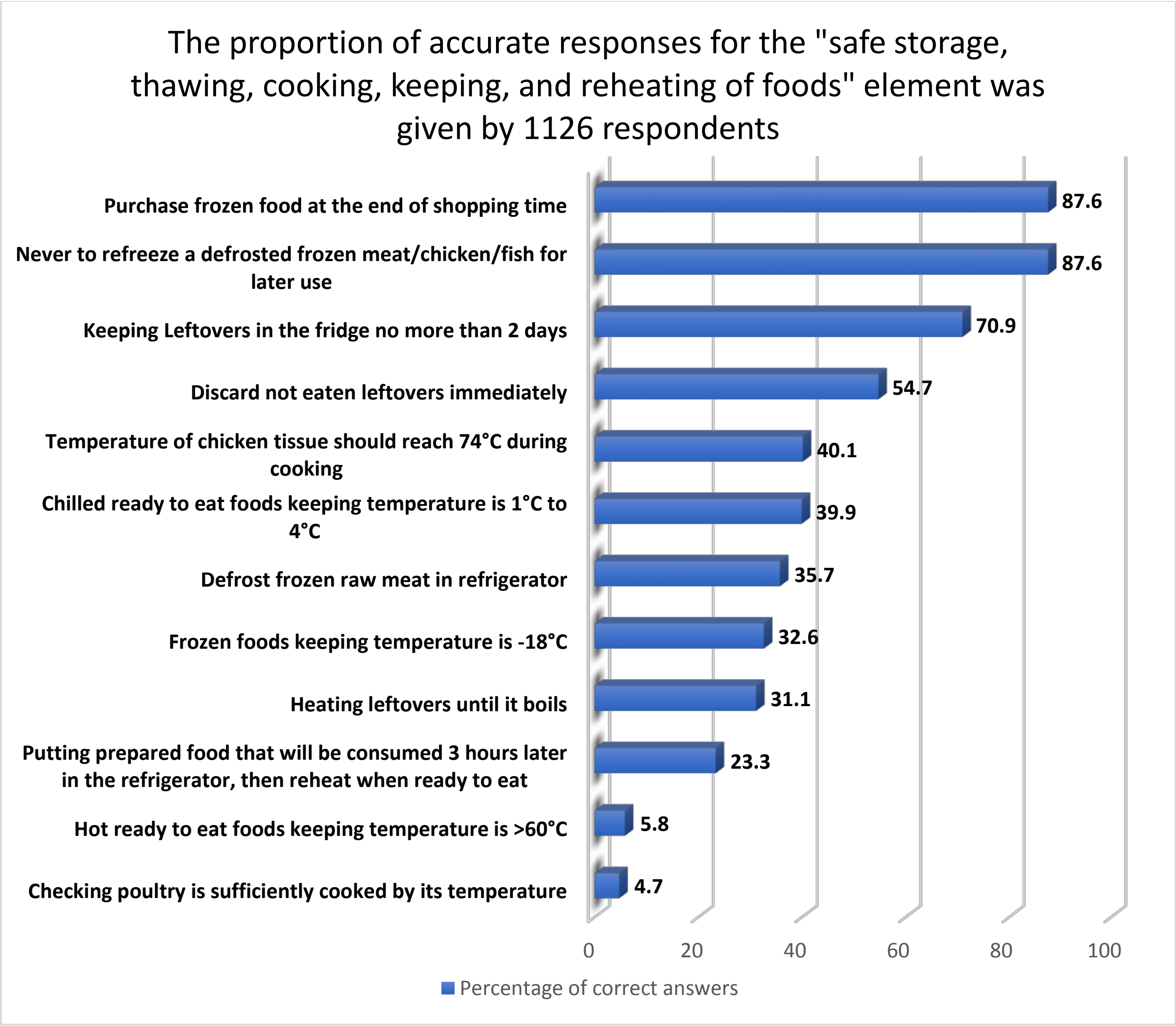
The proportion of accurate responses for the "safe storage, thawing, cooking, keeping, and reheating of foods" element was given by 1126 respondents
Purchase frozen food at the end of shopping time
87.6
Never to refreeze a defrosted frozen meat/chicken/fish for later use
87.6
Keeping Leftovers in the fridge no more than 2 days
70.9
Discard not eaten leftovers immediately
54.7
Temperature of chicken tissue should reach 74°C during cooking
40.1
Chilled ready to eat foods keeping temperature is 1°C to 4°C
39.9
Defrost frozen raw meat in refrigerator
35.7
Frozen foods keeping temperature is -18°C
32.6
Heating leftovers until it boils
31.1
Putting prepared food that will be consumed 3 hours later in the refrigerator, then reheat when ready to eat
23.3
Hot ready to eat foods keeping temperature is >60°C
5.8
Checking poultry is sufficiently cooked by its temperature
4.7
0
20
40
60
80
100
Percentage of correct answers

Fig 5

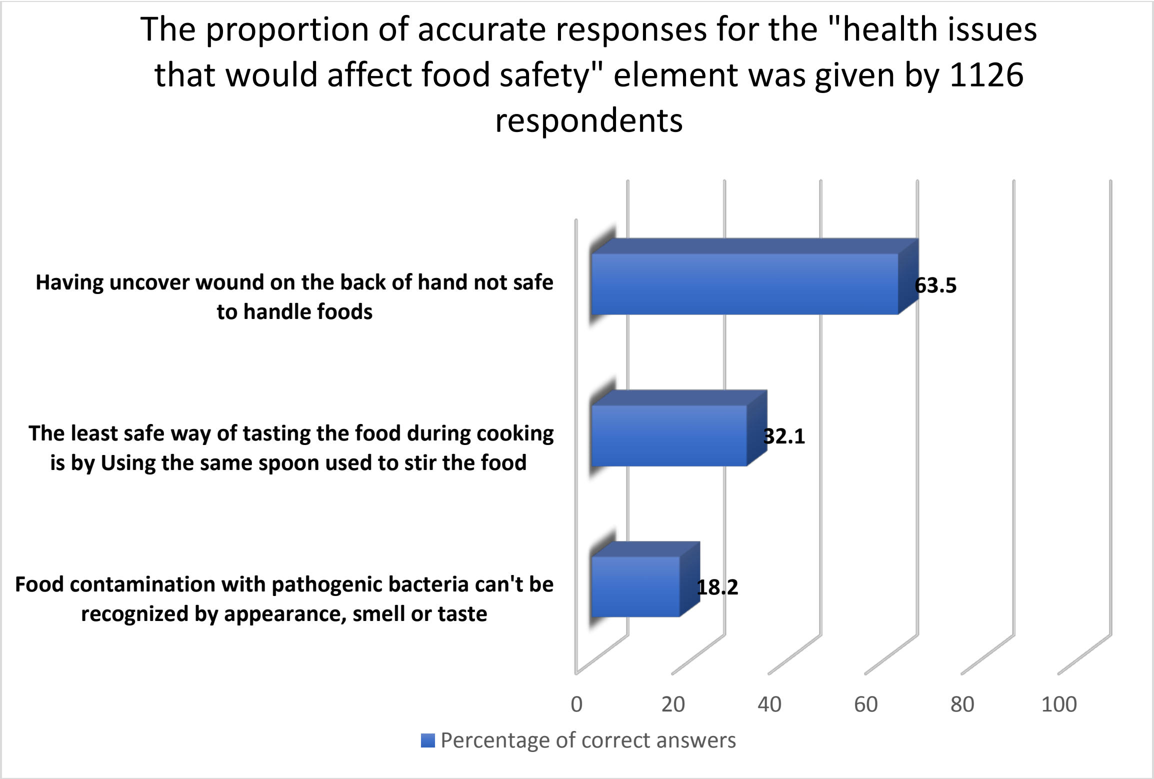
The proportion of accurate responses for the "health issues that would affect food safety" element was given by 1126 respondents
Having uncover wound on the back of hand not safe to handle foods
The least safe way of tasting the food during cooking is by Using the same spoon used to stir the food
Food contamination with pathogenic bacteria can't be recognized by appearance, smell or taste
63.5
32.1
18.2
0
20
40
60
80
100
Percentage of correct answers

Fig 6

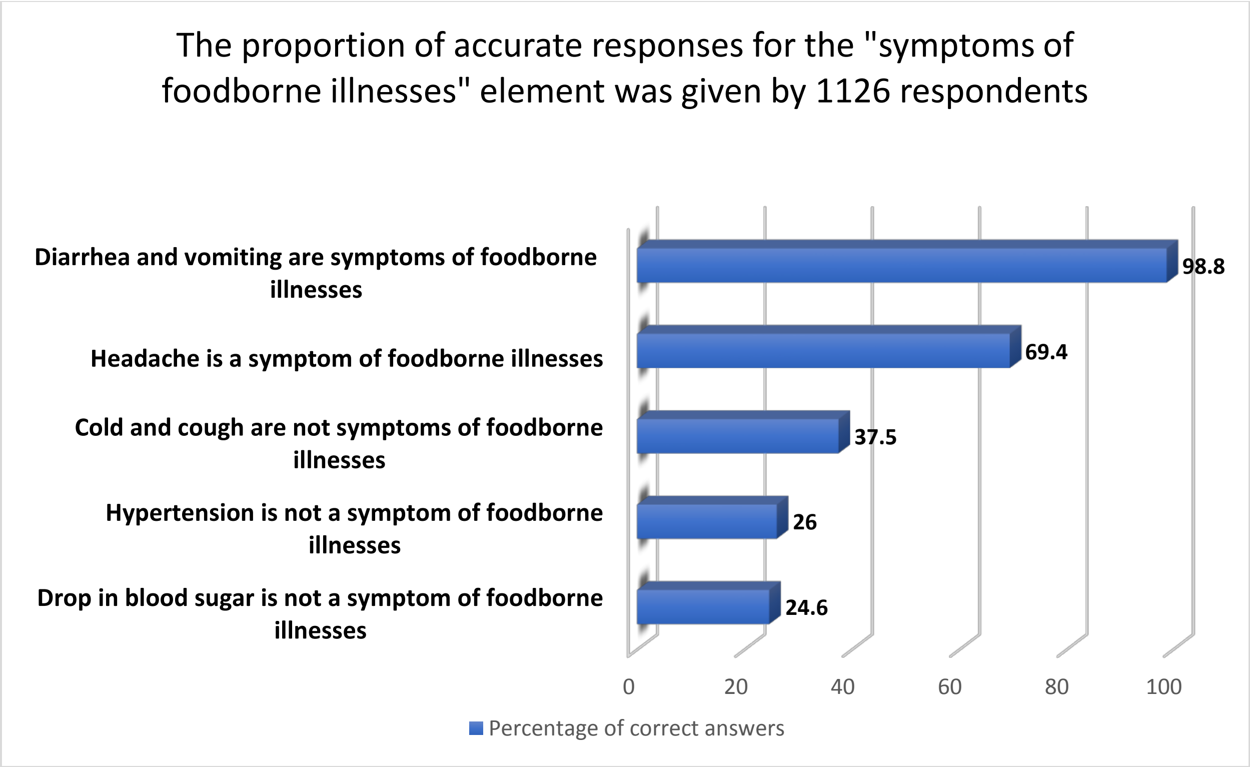
The proportion of accurate responses for the "symptoms of foodborne illnesses" element was given by 1126 respondents
Diarrhea and vomiting are symptoms of foodborne illnesses
Headache is a symptom of foodborne illnesses
Cold and cough are not symptoms of foodborne illnesses
Hypertension is not a symptom of foodborne illnesses
Drop in blood sugar is not a symptom of foodborne illnesses
98.8
69.4
37.5
26
24.6
0
20
40
60
80
100
Percentage of correct answers

Fig 7

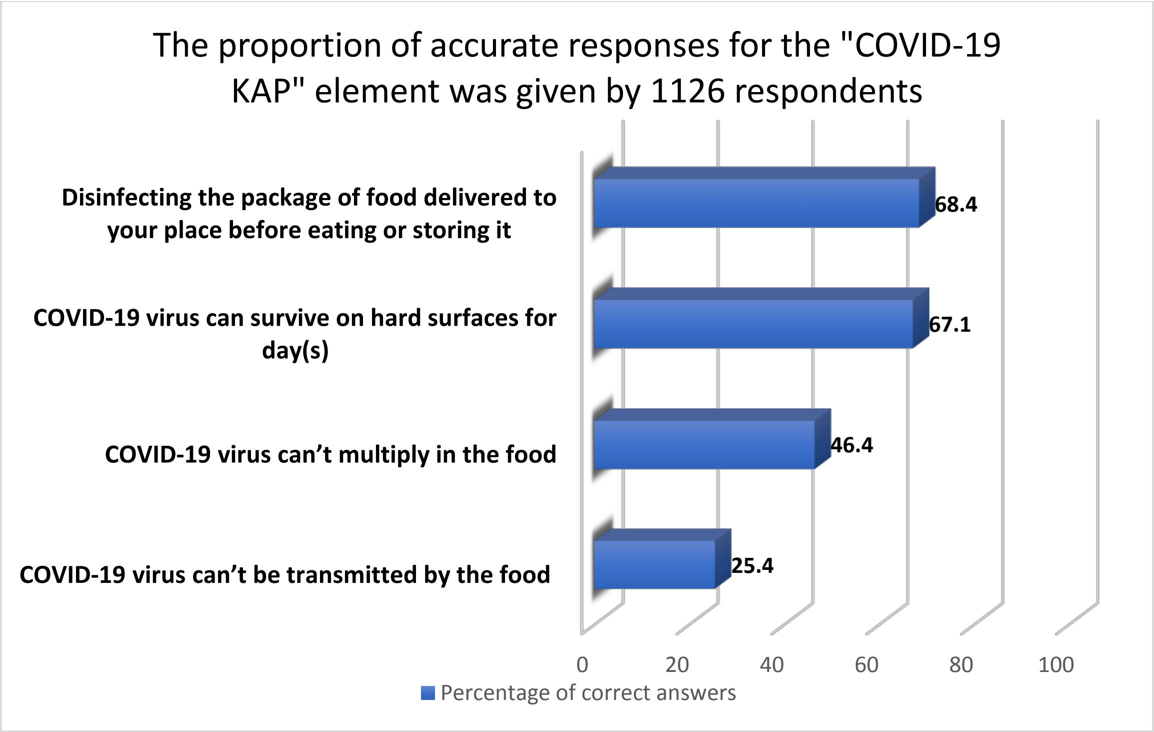
The proportion of accurate responses for the "COVID-19 KAP" element was given by 1126 respondents
Disinfecting the package of food delivered to your place before eating or storing it
68.4
COVID-19 virus can survive on hard surfaces for day(s)
67.1
COVID-19 virus can't multiply in the food
46.4
COVID-19 virus can't be transmitted by the food
25.4
0
20
40
60
80
100
Percentage of correct answers

Fig 8
