# Supplementary material for: Food safety knowledge, attitudes, and practices among Jordanian women handling food at home during COVID-19 pandemic
Source: PLoS One. 2023 Jul 7;18(7):e0288323. doi: 10.1371/journal.pone.0288323 (PMC10328301; doi:10.1371/journal.pone.0288323)
Supplement: Attachment — Submitted filename: PONE-D-22-35672 revised.docx [file pone.0288323.s002.docx]

**General Comments D-22-35672.**

The study, as a concept falls within the area of Food Safety KAP (knowledge, attitude, practises) and in the scope of PLOS, and specifically relating to Jordanian women.

Use of English in some cases, must be rechecked, for example, “subjects” you probably mean “humans” or individuals or home “consumers”. It is recommended to have a colleague whose English is his/her native language to improve the flow in the scientific presentation of the manuscript.

Parts of the Introduction section need to be filled in with more relevant to the study information, to justify the rationale and the need of the study.

Editing is required, in the Introduction section, and in general rechecking of words, expressions, proper use of verbal tenses, etc.

Data analysed followed standard statistical tools applied in such studies, and appropriately applied to the data processing.

Questionnaires well designed and types of questions being asked covered the target Food Safety KAP of home users, food handlers etc.

Results and Discussion, well presented and discussed.

**Specific Comments***

*Introduction*

**Page 3, Lines 56-58.** What about studies conducted in developing countries, related to women food handlers? You should provide examples, if available, related to women food handling, in the Middle East area, and what data showed.

**Page 3, Lines 56-59.** The EU studies, have these shown that the FBIOs are related to women food handlers? At home, be more specific. What it food prepared at home, or purchased from outlets other than home, or badly handled at home (that is poor personal hygiene). I do not see how this study relates to women food handlers… unless you provide more details. .

**Page 3, Line 63.** Did any of these studies, involve women? If any of those, did, what were the results? In summary? Expand on 1 or 2 studies…

**Page 3, Line 64.** Do you perhaps mean “regional” instead of local? And if local is meant in Jordan, in what is your study being different from previous? And what were the results from these studies? If you meant, regional like Lebanon etc… state and give a short paragraph on these results.

**Page 3, Lines 68-80.** Move this paragraph, in the beginning of the Introduction, best after line 41.

**Page 4, Line 73-76.** Delete lines… that is…starting from “Most to situation”.

**Page 4, Line 81.** Reword “Confront” to confronted… Use also past tense in other cases…in text, such as line 83; There “was” an urgent need…

**Pages 4-5, Line 83-87.** Too long paragraph and needs splitting in 2 shorter sentences.

**Page 5, Line 86.** “KAP” of what?

**Pages 4-5, Line 83-89.** Are there any other related data on women food handlers, regional or global, and what the data showed? Include a paragraph, if available, to justify the need of limited regional or global studies, on women, especially handling food in the Middle East.

*Materials and Methods*

**Page 5, Line 103.** Capitalize, “the Dep. of Statistics”

**Page 6, Line 122.** Rewrite in these two parts…. In the second and third part.

**Page 6, Line 126.** Reword “accurately assess what was meant to be assessed”. Why was a validation was conducted to those interviewed “face to face”?

**Page 6, Line 127.** Replace people with “women” and the word “subjects” throughout the manuscript, also Line Page 7, Lines 149, 150 etc.

**Page 7, Line 140.** Correct “Not” to not.

**Page 8, Lines 164-165.** Have other studies used a different % (60) value? And why did you decide to use the 60% value, based on 1 study only? Justify.

*Results and Discussion*

**Page 8, Lines 168.** In all, 1126 Jordanians. Reword better to “Jordanian” women…

**Page 8, Lines 172, 174.** Replace the word “polled” with voted, as this is a word used for *elections,* and in other sentences.

**Page 9, Lines 186-187.** “women food handlers in Jordan had inadequate knowledge, negative attitudes, and inappropriate practices”…. Is there a reason/reasons for that? Can you provide, why this is the case?

**Page 10, Line 205 .** Replace the word “discovered” with reported or corroborated.

**Reword Legends of Figs 2-8**. “Was given”.. Does not sound correct, perhaps reword with “given by”…

**Page 11, Line 229.** Did the “Canadian” study involve women food handlers? If yes, include it to validate your results.

**Page 11, Line 230-231. “**just 41.3 % **segregate** ready-to-eat food from raw food in the

231 refrigerator”, you meant here “**separate**” or reword.

**Page 12, Line 237.** Reword “Slovenian customers”.

**Page 12, Line 238**. Edit the word “About” to “about”.

**Page 12, Lines 240, 245.** Edit the word “identical” to “similar”, and food germs to “food bacteria”.

**Page 13, Line 255. “**These findings are **better** than “**tha**t”. Reword these 2 words.

**Page 13, Lines 259, 263, 268. “**of household women heat leftovers until **they are boiling**”. Reword. **Also,** “however, women in Lahore had a higher rate”. **Include** “of knowledge”. **Also,** and chicken tissue with “chicken meat”, and in Jordan's north… does not make sense. **Page 14, Line 284.** “tasting meals with the same utensils used to mix them”. Reword.

**Pages 14, 15…... Lines 388, 387, 383, 365, 360, 309, 301, 302, 299, 288, 289, 294.** “subjects and polled”. Reword.

**Page 18, Lines 358, 369**. “60th percentile”, should that be 60% or percentage?

**Page 20, Lines 378, 381, 385.** Perhaps “correlations”? instead of connections?

**Discussion: Well** supported, reasoned and being comprehensive.

**Tables:** Well presented, as well also Figures, with statistical evaluations included.

**Conclusions:** Adequate and well drawn.

*“Please also refer to the revised Pdf copy” for additional corrections.
