# Supplementary material for: Food safety knowledge, attitudes, and practices among Jordanian women handling food at home during COVID-19 pandemic
Source: PLoS One. 2023 Jul 7;18(7):e0288323. doi: 10.1371/journal.pone.0288323 (PMC10328301; doi:10.1371/journal.pone.0288323)
Supplement: Attachment — Submitted filename: Response to Reviewers PONE-D-22-35672 R1 25.05.2023.docx [file pone.0288323.s003.docx]

**Reviewer 1:**

It is a well-planned study to assess the food safety literacy among women food handlers at homes in Jordan. I have few questions.

**Comment 1:** How the sample size was calculated?

**Response:** The sample size was calculated based on the Jordanian Department of Statistics estimation of household number in Jordan, which is about 2,206,129 in 2020. This number was used in the equations mentioned in the **Materials and Methods page 6 lines 119-123.**

**Comment 2:** The designed questionnaire was in which language?

**Response:** The questionnaire was first design and written in English and then translated into Arabic which was then validated and distributed to the target population as mentioned in **section 2.3 Pilot Study page 7 line 144.**

**Comment 3**: As it is mentioned that questionnaire was distributed on social media, how it was ensured that it was rightly filled by a house woman?

**Response:** The questionnaire was distributed specifically in groups made only for housewives via social media platforms. **Page 8 line 159.**

**Comment 4**: Please mention the necessary actions that can be taken on the basis of findings of this study to improve the knowledge of food safety.

**Response:** Competent authorities should invest their efforts and energies in conceptualizing and constructing training programs to educate domestic food handlers about food safety practices and allow them to transform their learning into real-life settings to protect family members from foodborne illnesses **page 23 Lines 425-429**.

**Reviewer 2:**
The study, as a concept falls within the area of Food Safety KAP (knowledge, attitude, practises) and in the scope of PLOS, and specifically relating to Jordanian women.

**Comment** **1:** Use of English in some cases, must be rechecked, for example, “subjects” you probably mean “humans” or individuals or home “consumers”. It is recommended to have a colleague whose English is his/her native language to improve the flow in the scientific presentation of the manuscript.

**Response:** The manuscript has been edited by a native English speaker (Prof. Richard Holley, University of Manitoba, Canada. Email: rick.holley@umanitoba.ca).

**Comment** **2:** Parts of the Introduction section need to be filled in with more relevant to the study information, to justify the rationale and the need of the study.

**Response:** The manuscript has been revised to reflect this comment.

**Comment** **3:** Editing is required, in the Introduction section, and in general rechecking of words, expressions, proper use of verbal tenses, etc.

**Response:** The manuscript has been edited by a native English speaker (Prof. Richard Holley, University of Manitoba, Canada. Email: rick.holley@umanitoba.ca).

**Comment** **4:** Data analysed followed standard statistical tools applied in such studies, and appropriately applied to the data processing.

**Response**: Thank you.

**Comment** **5:** Questionnaires well designed and types of questions being asked covered the target Food Safety KAP of home users, food handlers etc.

**Response:** Thank you.

**Comment** **5:** Results and Discussion, well presented and discussed.

**Response:** Thank you.

**Specific Comments***

Introduction
**Comment 1:** Page 3, Lines 56-58. What about studies conducted in developing countries, related to women food handlers? You should provide examples, if available, related to women food handling, in the Middle East area, and what data showed.
**Response:** Yes, two cross-sectional studies was conducted on women food handlers. The first in Saudi Arabia on Saudi women to evaluate their food safety knowledge and practices at home and during shopping in 2023. The second study was conducted in Lahore to assess women’s Food safety knowledge, attitude, and food handling practices at home. The studies were added to the introduction **page 4 lines 74-83.**

**Comment 2:** Page 3, Lines 56-59. The EU studies, have these shown that the FBIOs are related to women food handlers? At home, be more specific. What it food prepared at home, or purchased from outlets other than home, or badly handled at home (that is poor personal hygiene). I do not see how this study relates to women food handlers… unless you provide more details. .
**Response:** Household outbreaks occurred when food is handled in improper way at home. This includes unhygienic manipulation of the ingredients by food handlers, cross-contamination, and temperature abuse. Please see **page 4 lines 84-86.**

**Comment 3**: Page 3, Line 63. Did any of these studies, involve women? If any of those, did, what were the results? In summary? Expand on 1 or 2 studies…
**Response:** No, none of these studies specified women as a target population. They assessed food safety KAP among food handlers and consumers in general. The lack of studies among women food handlers was one of the reasons we chose women as a population for our study. However, two studies conducted in developing countries related to women food handlers have been added to the introduction **page 4 lines 74-83.**

**Comment 4**: Page 3, Line 64. Do you perhaps mean “regional” instead of local? And if local is meant in Jordan, in what is your study being different from previous? And what were the results from these studies? If you meant, regional like Lebanon etc… state and give a short paragraph on these results.
**Response:** Local studies in Jordan were intended. In general, few studies examine food safety KAP in Jordan among different populations (universities, hospitals, restaurants, etc.). One study was conducted among Jordanian consumers. Our study is different from previous studies in the population studied, with only women participating in the current study and both women and men participating in the previous study. Also, there are differences in the food safety aspects covered in the two surveys. The current study covered almost all aspects of food safety, contrary to the previous study. Please see **page 5 lines 94-98.**

**Comment 5:** Page 3, Lines 68-80. Move this paragraph, in the beginning of the Introduction, best after line 41.
**Response:** Thank you for your suggestion. The paragraph was relocated to the beginning of the introduction as you requested.

**Comment 6:** Page 4, Line 73-76. Delete lines… that is…starting from “Most to situation”.
**Response:** The sentence was deleted upon your request.

**Comment 7:** Page 4, Line 81. Reword “Confront” to confronted… Use also past tense in other cases…in text, such as line 83; There “was” an urgent need…
**Response:** The words were corrected upon your request

**Comment 8:** Pages 4-5, Line 83-87. Too long paragraph and needs splitting in 2 shorter sentences.
**Response:** The paragraph was shortened and split into two sentences as you suggested.

**Comment 9:** Page 5, Line 86. “KAP” of what?
**Answer:** KAP of food handlers. A description was added to the text to avoid mess understanding.

**Comment 10:** Pages 4-5, Line 83-89. Are there any other related data on women food handlers, regional or global, and what the data showed? Include a paragraph, if available, to justify the need of limited regional or global studies, on women, especially handling food in the Middle East.
**Response:** Yes, these studies were added recently to the introduction **page 4 lines 74-83.**

Materials and Methods
**Comment 11**: Page 5, Line 103. Capitalize, “the Dep. of Statistics”

**Response:** Amended.

Page 6, Line 122. Rewrite in these two parts…. In the second and third part.
**Response:** Amended.

**Comment 12:** Page 6, Line 126. Reword “accurately assess what was meant to be assessed”. Why was a validation was conducted to those interviewed “face to face”?
**Response:** The sentence was rephrased. Face validity is about whether a test appears to measure what it's supposed to measure. Since this study relied on an online, self-administered questionnaire, pre-face validity is important to ensure that the questionnaire is understandable.

**Comment 13:** Page 6, Line 127. Replace people with “women” and the word “subjects” throughout the manuscript, also Line Page 7, Lines 149, 150 etc.
Page 7, Line 140. Correct “Not” to not.
**Response:** All the words were corrected; thank you for your suggestions.

**Comment 14:** Page 8, Lines 164-165. Have other studies used a different % (60) value? And why did you decide to use the 60% value, based on 1 study only? Justify

**Response:** The 60% passing rate (cut-off value) was determined by research done among Saudi mothers in 2018 (Ayaz, Priyadarshini, and Jaiswal, 2018), as mentioned in the text (**Page 9, lines 185-186**). This cut-off value was chosen because this population was not educated about food safety and had never attended food safety training. So, a higher cut-off value will not be suitable for assessing food safety KAP. However, women have a critical role in lowering the incidence of foodborne disease because they are primarily responsible for meal preparation at home. As a result, a lower cut-off value will not provide an accurate evaluation for this population.

Results and Discussion

**Comment 15:** Page 8, Lines 168. In all, 1126 Jordanians. Reword better to “Jordanian” women…

**Response:** Amended.

Page 8, Lines 172, 174. Replace the word “polled” with voted, as this is a word used for elections, and in other sentences.
**Response:** Amended.

**Comment 16:** Page 9, Lines 186-187. “women food handlers in Jordan had inadequate knowledge, negative attitudes, and inappropriate practices” …. Is there a reason/reasons for that? Can you provide, why this is the case?
**Response:** Women food handlers in Jordan had inadequate knowledge, negative attitudes, and inappropriate practices because they were not educated about food safety and had never attended food safety training.

**Comment 17**: Page 10, Line 205 . Replace the word “discovered” with reported or corroborated.
Reword Legends of Figs 2-8. “Was given”.. Does not sound correct, perhaps reword with “given by”…
**Response:** Thank you for your suggestion. The words were changed upon your request

**Comment 18:** Page 11, Line 229. Did the “Canadian” study involve women food handlers? If yes, include it to validate your results.
**Response:** No, the study conducted in Canada did not specify women as a target population.

**Comment 19:** Page 11, Line 230-231. “just 41.3 % segregate ready-to-eat food from raw food in the
231 refrigerator”, you meant here “separate” or reword.
Page 12, Line 237. Reword “Slovenian customers”.

Page 12, Line 238. Edit the word “About” to “about”.

Page 12, Lines 240, 245. Edit the word “identical” to “similar”, and food germs to “food bacteria”.

Page 13, Line 255. “These findings are better than “that”. Reword these 2 words.
Page 13, Lines 259, 263, 268. “of household women heat leftovers until they are boiling”. Reword. Also, “however, women in Lahore had a higher rate”. Include “of knowledge”. Also, and chicken tissue with “chicken meat”, and in Jordan's north… does not make sense.

Page 14, Line 284. “tasting meals with the same utensils used to mix them”. Reword.

Pages 14, 15…... Lines 388, 387, 383, 365, 360, 309, 301, 302, 299, 288, 289, 294. “subjects and polled”. Reword.

Page 18, Lines 358, 369. “60th percentile”, should that be 60% or percentage?

Page 20, Lines 378, 381, 385. Perhaps “correlations”? instead of connections?
**Response:** The manuscript has been modified to reflect these comment.

**Comment:** Discussion: Well supported, reasoned and being comprehensive.

**Response:** Thank you.

**Comment:** Tables: Well presented, as well also Figures, with statistical evaluations included.

**Response:** Thank you.

**Comment:** Conclusions: Adequate and well drawn.

**Response:** Thank you.

**Comment:** *“Please also refer to the revised Pdf copy” for additional corrections.

**Response:** The manuscript has been revied to reflect the comments on the pdf file

**Reviewer 3:**
Some minor corrections:
**Comment 1:** page 9, line 191, some references were mentioned directly. Corrections should be done for all the references.
**Response:** All references were revised and corrected. Thank you.

**Comment 2:** Page 11, line 217, Figures mentioned but there were no figures in all the manuscript.

**Response:** Yes, the figures captions were mentioned in the manuscript, but the figures were submitted in a separate file according to PLOS guidelines in a file named "Food safety KAP among households’ figures”. I do not know whether it is a technical issue that the file was unavailable for you to review, so please accept our apologies.
